# Fluctuations in nitrate availability impact cytokinin biosynthesis through histone modifications of *IPT3* in *Arabidopsis* roots for growth acclimation

**DOI:** 10.1016/j.xplc.2025.101531

**Published:** 2025-09-23

**Authors:** Fanny Bellegarde, Olivia Tjahjono, Mika Yoshino-Kida, Takatoshi Kiba, Miki Shibutani, Mei Kuriyama, Louis J. Irving, Mikiko Kojima, Kazuki Miyata, Hitoshi Sakakibara

**Affiliations:** 1 Graduate School of Bioagricultural Sciences, Nagoya University, Aichi 464-8601, Japan; 2 Institute for Advanced Research, Nagoya University, Aichi 464-8601, Japan; 3 Institute of Life and Environmental Sciences, University of Tsukuba, Tennodai 1-1-1, Tsukuba 305-8577, Japan; 4 RIKEN Center for Sustainable Resource Science, Tsurumi, Yokohama 230-0045, Japan

**Keywords:** *Arabidopsis*, cytokinin biosynthesis, transcriptional regulation, histone modification, nitrate fluctuation, growth acclimation

## Abstract

Nitrate availability in soil is highly variable and often a limiting factor for crop growth. Plants must acclimate rapidly to these fluctuations. The phytohormone cytokinin (CK) plays a pivotal role in nitrate signaling as a secondary growth regulator. However, the mechanisms that regulate CK action in response to fluctuating nitrate levels remain poorly understood. Here, we show that chromatin modification of *IPT3*, a key gene in CK biosynthesis, is crucial for growth acclimation to variable nitrate supply. Transcriptional regulation of *IPT3* drives CK output in response to nitrate availability, thereby balancing root and shoot growth. This rapid and dynamic regulation is mediated by two antagonistic histone H3 modifications: trimethylation of lysine 27 of histone H3 (H3K27me3) and H3K4me3. Using mutants defective in the deposition or removal of these modifications, we identify several chromatin effectors involved in these processes and confirm that nitrate-driven chromatin dynamics fine-tune CK biosynthesis. Our findings provide insights into the epigenetic regulatory mechanisms underlying CK biosynthesis and open new avenues faor enhancing plant acclimation to fluctuating nutrient environments.

## Teaser

This study unravels the temporal dynamics of plant responses to nitrate fluctuations, the relationship between growth acclimation and cytokinin biosynthesis, and the mechanisms underlying its transcriptional regulation. In particular, *IPT3* is dynamically regulated by nitrate fluctuation, thereby controlling cytokinin output and balancing root and shoot growth. The transcriptional regulation of *IPT3* is mediated by the interplay between H3K4me3 and H3K27me3 in response to nitrate availability, demonstrating that nitrate-driven chromatin dynamics fine-tune cytokinin biosynthesis to facilitate growth acclimation.

## Introduction

Nitrate is a major nitrogen source for plants in both natural and agricultural ecosystems (Crawford and Forde, 2002). It is formed in soil through the conversion of ammonia from plant and animal residues or fertilizers into nitrate by soil bacteria. However, nitrate is highly mobile and readily leached by rainfall, and its concentration in soil fluctuates considerably. Plant roots are therefore often exposed to variable nitrate levels, which can significantly impact plant growth and thus, crop productivity. To cope with this temporal heterogeneity, plants have evolved mechanisms that sense changes in nutrient availability and enable rapid growth acclimation.

Nitrate perception triggers both local and systemic signals that coordinate uptake with growth (Alvarez et al., 2019). At the local level, nitrate uptake induces calcium influx, which activates calcium-sensor protein kinases. These kinases phosphorylate NIN-LIKE PROTEIN 7 (NLP7), a master transcriptional activator of nitrate responses (Liu et al., 2017). NLP7 then induces primary nitrate response genes, often referred to as "sentinel genes" (Krouk et al., 2010), which are involved in nitrate transport (e.g., *NRT1.1* and *NRT2.1*) and assimilation (e.g., *NIA1* and *NIR1*) (Scheible et al., 2004; Liu et al., 2017). For systemic nitrate-dependent growth signaling, cytokinins (CKs), a class of plant hormones, play a pivotal role in root-to-shoot communication (Sakakibara, 2021). Among CKs, *N*^*6*^-(Δ^2^-isopentenyl)adenine (iP) and *trans*-zeatin (tZ) are the major forms, with tZ riboside (tZR) being the dominant xylem-transported form (Ko et al., 2014; Osugi et al., 2017). Nitrate signaling induces key CK biosynthesis genes such as *ADENOSINE PHOSPHATE-ISOPENTENYLTRANSFERASE* (*IPT*), which produces iP riboside (iPR) phosphate (iPRP). CYP735A2 then catalyzes the conversion of iPRP to tZ riboside phosphate (tZRP). Among the *IPT* gene family, *IPT3* is a key determinant of nitrate-dependent CK biosynthesis (Takei et al., 2004). Conversely, *IPT3* and *CYP735A2* are repressed under nitrate starvation (Ramireddy et al., 2014). Despite these insights, relatively few studies have analyzed the role of transcription factors in regulating CK biosynthesis.

Accumulating evidence suggests that NLP7 acts as an effector of nitrate-mediated induction of *IPT3*. This induction is impaired in the *nlp7* mutant (Abualia et al., 2022), but only reduced in nitrate reductase mutants (Wang et al., 2004). In addition, nitrate-induced increases in seedling tZ levels are modulated by NLP and NIGT1 through the regulation of *CYP735A2* (Maeda et al., 2018). Despite these insights, the mechanisms by which plants control CK production to facilitate growth acclimation under nitrogen fluctuations remain largely unexplored.

The accessibility of DNA information is controlled by chromatin. Histone methylation appears to have an essential role in regulating gene expression, including responses to abiotic stresses (Shi et al., 2024). The deposition or removal of histone methylation is dynamically regulated by histone methyltransferases and demethylases, respectively. POLYCOMB REPRESSIVE COMPLEX 2 (PRC2), which includes methyltransferases such as CURLY LEAF (CLF) and SWINGER during vegetative growth, mediates the trimethylation of lysine 27 of histone H3 (H3K27me3) to stably repress genes with low or no expression (Bemer and Grossniklaus, 2012). Demethylation of H3K27me3 is catalyzed by JUMONJI (JMJ) family proteins, including JMJ11/EARLY FLOWERING 6 (ELF6), JMJ12/RELATIVE OF EARLY FLOWERING 6 (REF6), and JMJ13 (Lu et al., 2011; Gan et al., 2015). In contrast, trithorax group proteins, such as ARABIDOPSIS TRITHORAX-LIKE (ATX) and ATX-RELATED members, promote gene activation through H3K4 trimethylation (Alvarez-Venegas et al., 2003). Conversely, H3K4me3 demethylation is mediated by members of the KDM5/JARID subfamily, such as JMJ14, which regulates flowering time (Lu et al., 2010) and responses to environmental stress (Cui et al., 2021; Sun et al., 2021). While these antagonistic modifications are well characterized in fine-tuning the expression of genes involved in development and responses to abiotic stresses (e.g., heat, cold, and salt), their roles in plant responses to nutritional changes (Secco et al., 2017)—particularly under fluctuating nitrate availability—remain largely unknown.

In this study, we investigated how CK biosynthesis contributes to plant growth acclimation under fluctuating nitrate conditions, focusing on the transcriptional regulation of CK biosynthesis genes. We show that *IPT3* regulation plays a crucial role in maintaining the root–shoot growth balance in response to nitrate availability. The transcriptional regulation of *IPT3* is predominantly influenced by dynamic changes in H3K27me3 and H3K4me3, representing repressive and activating histone marks, respectively. Using mutants defective in the deposition or removal of these modifications, we demonstrate that the chromatin state of *IPT3* changes dynamically in response to nitrate fluctuations, thereby impacting CK output. This study provides an integrated view of how plants adapt to fluctuating nutrient environments by linking chromatin-based transcriptional regulation to growth responses. It also lays the foundation for a deeper understanding of how nitrate availability reshapes chromatin landscapes to regulate plant growth and development.

## Results

### Nitrate fluctuation controls CK biosynthesis and transport

**Figure 1:**
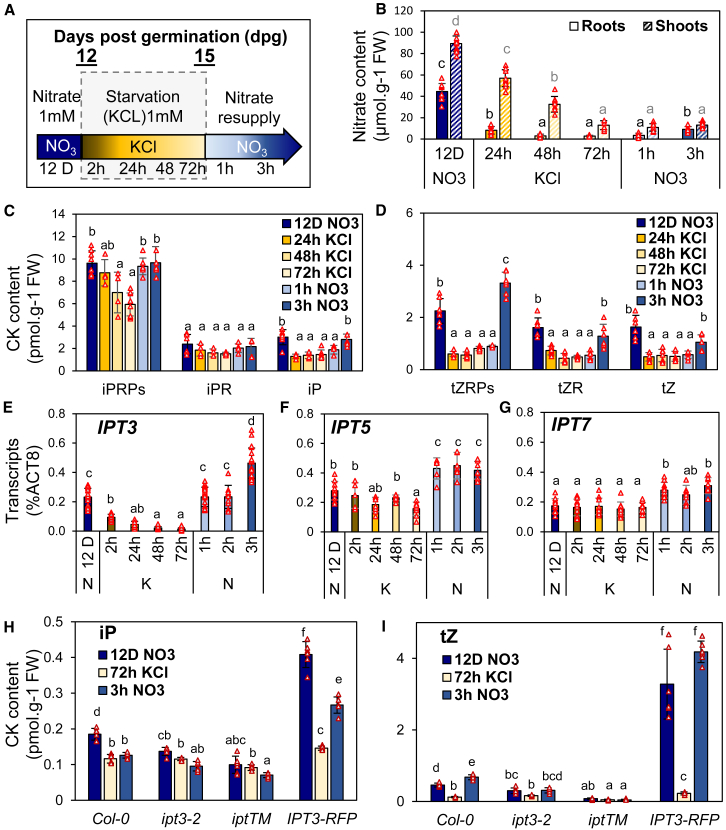
Variation in nitrate availability strongly impacts root CK content and parallels *IPT3* transcript profiles. **(A)** Schematic representation of the nitrate fluctuation kinetics used in most experiments with 12- to 15-day-old plants grown hydroponically and the color code for each time point: shades of blue for nitrate and shades of yellow for starvation. **(B)** Nitrate content in roots (solid colors) and shoots (striped colors) during the nitrate fluctuation time course (see also Supplemental Figure 1). **(Cand D)** Root CK quantification of iP and its precursors **(C)** and tZ and its precursors **(D)** during the nitrate fluctuation time course. **(E–G)** Transcript levels of *IPT3***(E)**, *IPT5***(F)**, and *IPT7***(G)** during nitrate fluctuation. **(Hand I)** Root CK quantification of iP **(H)** and tZ **(I)** during the nitrate fluctuation time course in *ipt* mutants and the complementation line (IPT3-RFP) (see also Supplemental Figure 2). Data are presented as mean ± SD. *N* = 7–8 **(B)**, 4–6 **(C** and **D)**, 15–20 **(E)**, 5–10 **(F)**, 8–10 **(G)**, and 4–6 **(H** and **I)** individual plants (red triangles) grown hydroponically. One-way ANOVA was performed, and letters indicate significant differences based on Tukey’s HSD test, *p* < 0.05.

To gain insight into the role of CK in growth acclimation to fluctuating nitrate availability, we conducted time-course experiments with *Arabidopsis* grown hydroponically. Plants were supplied with 1 mM nitrate (KNO_3_) for 12 days post-germination (dpg), followed by 3 days of nitrate starvation (1 mM KCl), and then resupplied with 1 mM nitrate (Figure 1A).

We first characterized the physiological responses to nitrate fluctuations by quantifying nitrate, chlorophyll, and CK content as well as gene expression (Figure 1; Supplemental Figure 1). Under nitrate-sufficient conditions, nitrate content was higher in shoots than in roots, indicating predominant storage in shoots (Figure 1B). During starvation, root nitrate levels dropped rapidly and were nearly depleted after 48 h, whereas shoot levels declined more gradually. Total chlorophyll content decreased after 72 h, marking the onset of visible nitrate deficiency (Supplemental Figure 1A). Plants grown under constant nitrate for 12 or 15 dpg showed similar nitrate and chlorophyll levels (Supplemental Figures 1B and 1C), confirming that the observed effects were due to nitrate fluctuations rather than growth differences. Root CK quantification revealed a pattern similar to that of nitrate content, particularly for the active CK forms. iPRP was the most abundant iP precursor; although it declined significantly after 24 h, its level remained higher than that of other forms even after 72 h of deficiency, whereas iPR was less responsive (Figure 1C). By contrast, iP, tZ, and their precursors dropped to basal levels within 24 h (Figures 1C and 1D). This early decline in CK content suggests that roots responded to nitrate depletion, triggering active repression. Analysis of *IPT* gene expression profiles revealed that changes in CK levels correlate with *IPT3* transcription—*IPT3* repression occurred rapidly within 2 h of starvation and reached basal levels by 24 h; *IPT5* repression occurred later at 72 h, while *IPT7* remained unaffected (Figures 1E–1G). Upon nitrate resupply, root nitrate levels increased significantly within 3 h, with an influx surge detectable after just 2 h (Supplemental Figure 1D). Shoot nitrate levels did not increase within this period, but chlorophyll content began to recover (Supplemental Figure 1A). These results suggest that absorbed nitrate was prioritized for its assimilation, enabling chlorophyll recovery before accumulation in roots. Nitrate perception in roots triggered sentinel gene expression (Supplemental Figures 1E–1G) and the rapid induction of *IPT3*, -*5*, and -*7* within 1 h (Figures 1E–1G). *IPT3* exhibited a two-phase induction: an initial increase within 15 min, which remained stable until 2 h, followed by a further rise after 3 h as nitrate levels continued to increase (Figure 1; Supplemental Figure 2A).

To investigate the role of CK produced by IPT3 in plant growth and development, we generated an IPT3-tagRFP (IPT3-RFP) line under the control of the *IPT3* promoter in the *ipt3 ipt5 ipt7* triple mutant (*iptTM*) background. Unlike the mutants, which showed low *IPT3* expression, IPT3-RFP exhibited higher expression on nitrate than the wild type (WT) but was repressed after 3 days of nitrogen starvation (Supplemental Figures 2A and 2B). Upon nitrate resupply, *IPT3* expression in IPT3-RFP was induced similarly to that in the WT, beginning at 15 min and increasing significantly after 30 min. CK quantification revealed higher iP- and tZ-type CK levels in IPT3-RFP compared with WT, whereas the mutants showed lower levels (Figures 1H and 1I; Supplemental Figures 2C–2F). After 3 days of starvation, CK levels in WT and IPT3-RFP decreased to those observed in *ipt3-2* and *iptTM*. These results suggest that IPT3-RFP functions as an *IPT3-*overexpressing line while retaining proper regulatory control. The RFP signal, reflecting IPT3 protein levels, localized specifically to phloem companion cells and followed *IPT3* transcriptional dynamics in response to nitrate fluctuations (Supplemental Figures 3A–3C). Across root regions, repression during starvation occurred more rapidly from the middle to root tips than in upper roots (Supplemental Figures 3D and 3E), suggesting that *IPT3* downregulation occurs faster in younger tissues, while expression is maintained in older root zones.

All genotypes showed a notable effect on tZ-type CKs, especially after nitrate resupply (Figure 1I; Supplemental Figures 2E and 2F). To examine whether IPT3 regulates the expression of *CYP735A2* and *ABCG14,* a major root-to-shoot CK transporter, we analyzed their expression under fluctuating nitrate conditions (Supplemental Figures 2G–2J). In WT plants, both genes were suppressed during nitrogen starvation and induced within 15 min of nitrate resupply. In *iptTM*, induction was delayed, whereas *ipt3-2* showed little effect on their early induction. However, in both *ipt3-2* and *iptTM*, the expression of the two genes was strongly reduced after 3 h of resupply, suggesting that the sustained induction of *CYP735A2* and *ABCG14* by nitrate is dependent on IPT3-derived CKs (Supplemental Figures 2G–2I). IPT3-RFP restored the normal expression of *CYP735A2* and *ABCG14*, with transcript levels exceeding those of WT plants after prolonged nitrate exposure (12 days) and after 3 h of resupply (Supplemental Figures 2H–2J). Specifically, *CYP735A2* expression was twice as high after prolonged nitrate exposure and fourfold higher after 3 h of resupply in IPT3-RFP compared with WT (Supplemental Figures 2H–2J), consistent with elevated CK accumulation (Figure 1I). In addition, both genes were induced by prolonged CK treatment even in the absence of nitrate, in contrast to *IPT3* and nitrate sentinel genes, which were unresponsive—except for *NRT1.1*, which was repressed at high tZ concentrations (Supplemental Figure 4). These findings reveal that CK-mediated induction of *CYP735A2* and *ABCG14* is independent of nitrate signaling (other than CK) and suggest that *CYP735A2* and *ABCG14* are rapidly (within minutes) regulated by nitrate and later by CK accumulation, with *IPT3* playing a central role in coordinating this process.

### IPT3 plays a key role in plant growth acclimation to nitrate fluctuation

**Figure 2:**
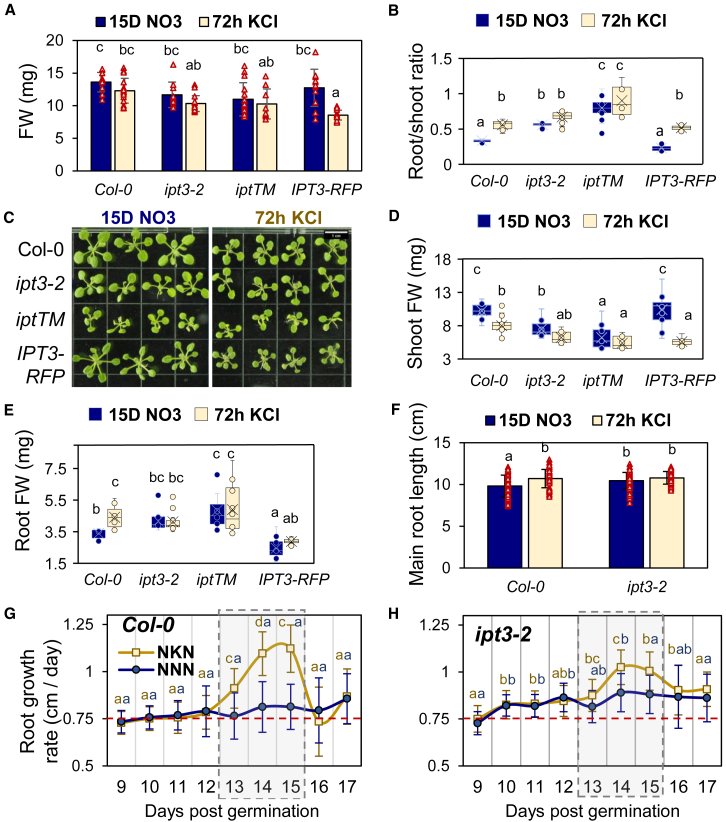
*IPT3* maintains a balance between root and shoot growth by restricting nitrate-dependent primary root growth. **(A–E)** Growth phenotypes of 15-day post-germination (dpg) plants grown hydroponically, including total fresh weight (FW) **(A)**, the root/shoot FW ratio **(B)**, representative shoot phenotypes of 3–4 plants per genotype **(C)** (scale bar: 1 cm), and corresponding shoot **(D)** and root **(E)** FW. All boxplots show inner and outlier points used to calculate the mean (cross); quartiles were calculated by the exclusive median (see also Supplemental Figures 5 and 6). **(F–H)** Root growth of plants grown on Petri dishes with the same medium supplemented with agar, showing primary root length **(F)** and growth rate comparison between WT **(G)** and *ipt3-2***(H)** under constant (NNN) or fluctuating (NKN) nitrate conditions. The treatment period (“K” for KCl or “N” for NO_3_) between 12 and 15 dpg is highlighted by a gray rectangle. The rate of primary root growth corresponds to the daily increase in root length between 9 and 17 dpg (see also Supplemental Figure 7). Data are presented as mean ± SD. *N* = 12–15 **(A**, **B**, **D**, and **E)**, 42–57 **(F)**, and 35–70 **(G** and **H)** individual plants. Letters indicate significant differences according to one-way ANOVA followed by Tukey’s HSD test, *p* < 0.05. For **(G) and (H)**, one-way ANOVA was performed at each time point, with blue letters indicating the NNN condition and orange letters indicating the NKN condition. A red dashed line was added as a visual aid to compare genotypes.

Next, we examined how differential CK production influences growth by comparing the fresh weight (FW) of 15-day-old plants after 72 h of starvation or continuous nitrate supply (Figure 2). In the WT, 3 days of starvation did not affect total seedling FW (Figure 2A) but led to a higher root/shoot ratio (Figure 2B), attributed to reduced shoot FW and increased root FW (Figures 2C–2E). Interestingly, *ipt3-2* showed no difference in the total mass or root/shoot ratio between treatments, with statistical grouping aligning it with starved WT plants (Figures 2B, 2D, and 2E). This suggests that *ipt3-2* exhibits a starvation-like growth pattern regardless of nitrate availability. The phenotype correlated with CK levels, suggesting the importance of *IPT3* in regulating CK production and maintaining a balance between root and shoot growth in response to nitrate. In *iptTM*, the shift in root/shoot ratio was even more pronounced; however, IPT3-RFP rescued the phenotype under nitrate supply. In this background, shoot FW was restored, whereas root FW decreased (Figures 2D and 2E).

We repeated the experiment with additional time points using agar plates and confirmed the phenotype at 15 dpg (Supplemental Figures 5A–5F). The root/shoot ratio showed a pattern similar to that of hydroponic culture, though differences were not always statistically significant. Compared with hydroponics, where plants had higher biomass, starvation-induced root growth stimulation was observed in the WT and IPT3-RFP on agar plates. Also, shoot FW showed a notable reduction in WT and IPT3-RFP after starvation. The *ipt3-2* phenotype became more pronounced after 5 days of nitrate resupply (20 dpg) (Supplemental Figures 5G–5K), with total FW being significantly lower than that of WT, mainly due to reduced shoot FW. These results suggest that *IPT3* induction after starvation is important for promoting shoot growth.

Similar growth profiles were observed across all time points on Petri dishes under constant nitrate conditions (Supplemental Figures 5 and 6). The earliest detectable effect of *ipt3-2* on biomass appeared at 12 dpg, with an increase in root FW, whereas shoot FW remained unaffected (Supplemental Figures 6A–6E). This increase was not due to more lateral roots but correlated with the elongation of primary roots (Supplemental Figures 6F and 6G). At 15 dpg (Figure 2F), WT plants showed significant primary root elongation in response to starvation; however, this was not evident in *ipt3-2*. These results suggest that the higher root FW observed in *ipt3-2* under nitrate conditions is primarily driven by increased root length. We then estimated primary root growth rates (Figures 2G and 2H; full profile in Supplemental Figure 7). Consistent with their longer roots, *ipt3-2* seedlings exhibited a significantly higher growth rate than WT at 10 dpg, whereas IPT3-RFP showed a marked reduction as early as 1 dpg (Supplemental Figure 7). Within 24 h of starvation (at 13 dpg), both *ipt3-2* and WT showed increased root growth rates, accompanied by decreases in active CK (Figures 1C and 1D). Conversely, 24 h after nitrate resupply (16 dpg), WT plants exhibited a sharp reduction in root growth rate, which was significantly attenuated in *ipt3-2* (Figures 2G and 2H) but more pronounced in IPT3-RFP (Supplemental Figure 7). Thus, decreased CK content was associated with increased root growth, and CK accumulation reduced root growth rates. These results highlight the rapid acclimation of root growth to nitrate fluctuations within 24 h, a process that depends on root CK levels.

Root growth is closely linked to meristem size, which is regulated by phytohormones including CK (Dello Ioio et al., 2007). To assess the role of *IPT3*, we analyzed root meristem size in *ipt3-2* by counting the number of cortical cells in the meristematic zone (Supplemental Figure 8). Consistent with primary root growth profiles, *ipt3-2* mutants had more cortical cells than WT plants at all time points under constant nitrate conditions (Supplemental Figure 8), whereas IPT3-RFP showed the opposite trend. Starvation increased cortical cell numbers in WT and IPT3-RFP but not in *ipt3-2*, which already had elevated cell counts under nitrate. Statistical analysis linked starved WT plants with *ipt3-2* across conditions (Supplemental Figure 8B), highlighting the essential role of *IPT3* in nitrate-dependent regulation of root meristem size, which in turn controls primary root elongation.

Overall, these results reveal that fluctuations in nitrate availability affect the balance between root and shoot growth, with CK acting as a central regulator. This regulation depends largely on *IPT3* transcription: (1) its repression is essential for starvation-induced primary root growth and meristem expansion, and (2) its induction is necessary for inhibiting primary root growth and stimulating shoot growth after nitrate resupply.

### Histone modifications correlate with nitrate-dependent regulation of *IPT3* transcription

**Figure 3:**
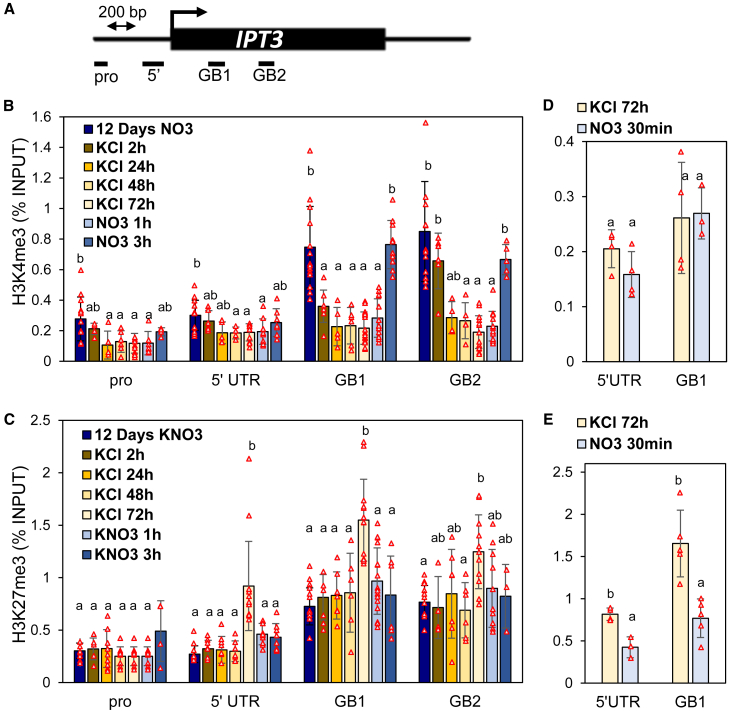
Fluctuation in nitrate availability alters the chromatin profile of *IPT3* in roots. **(A)** Schematic representation of the *IPT3* locus and four regions analyzed by ChIP–qPCR. Pro, promoter; 5′, inside the 5′ UTR; GB, gene body. **(Band C)** Impact of nitrate fluctuation (12–15 dpg) on enrichment of H3K4me3 **(B)** and H3K27me3 **(C)** at the *IPT3* locus (as shown in **A**) relative to input. **(Dand E)** Impact of short-term nitrate resupply on enrichment of H3K4me3 **(D)** and H3K27me3 **(E)** at the *IPT3* locus. Data are presented as mean ± SD. *N* = 5–13 **(B** and **C)** and 4–5 **(D** and **E)** biologically independent samples (red triangles). One-way ANOVA was performed for each region, and letters indicate significant differences according to Tukey’s HSD test, *p* < 0.05 (see also Supplemental Figures 9 and 10).

During plant growth and development, the balance between H3K4me3 and H3K27me3 strongly influences transcriptional programs (Engelhorn et al., 2014; Xiao et al., 2016). To determine whether these histone marks regulate *IPT3* transcription in response to fluctuating nitrate, we focused on root responses. Analysis of the *IPT3* locus revealed significant changes in both marks, particularly in the 5′ UTR and gene body, while the promoter was less affected (Figures 3A–3C). Under nitrate-sufficient conditions, when *IPT3* was expressed, its locus was enriched in the active mark H3K4me3 (Figure 3B). Upon nitrate starvation, *IPT3* repression occurred within 2 h and was marked by a reduction in H3K4me3—first in the gene body and later, after 24 h, in the promoter and 5′ UTR. H3K27me3 deposition appeared only after 3 days of starvation, across all regions except the promoter (Figure 3C). When nitrate was resupplied, *IPT3* expression was rapidly induced within 30 min (Supplemental Figure 2), without concurrent H3K4me3 deposition but accompanied by a reduction in H3K27me3 (Figures 3D and 3E). H3K4me3 deposition became evident only after 3 h of resupply (Figure 3B), coinciding with the increase in root nitrate content and the second phase of *IPT3* induction (Figure 1; Supplemental Figure 2).

The H3K27me3 and H3K4me3 enrichment at the control genes *LEC2* and *ACT7* showed no response to nitrate fluctuation (Supplemental Figure 9), suggesting that it is nitrate fluctuation, rather than other developmental cues, that triggers histone modifications on *IPT3*. In addition, no differences were observed under constant nitrate between 12 and 15 dpg (Supplemental Figure 10). These results confirm that nitrate availability directly and dynamically influences the chromatin state of the *IPT3* locus.

### Nitrate starvation leads to H3K4me3 demethylation and H3K27me3 deposition at the *IPT3* locus

To further investigate the role of histone modification dynamics in *IPT3* transcription, we analyzed mutants impaired in the deposition or removal of both H3K4me3 and H3K27me3. Our analysis focused on gene body region 1 (Figure 3A), which showed the strongest and most rapid response to nitrate fluctuations.

Based on Figure 3, we hypothesized that nitrate starvation rapidly decreases H3K4me3 levels through the action of H3K4me3 demethylases, thereby repressing *IPT3* transcription. Meanwhile, H3K27 trimethylation by H3K27 methyltransferases sustains *IPT3* repression. To identify the chromatin effectors involved, we analyzed the expression profiles of H3K4me3 demethylases and H3K27 methyltransferases during N starvation (Supplemental Figure 11). Among the KDM5 subfamily, *JMJ14* showed the highest expression and sensitivity to starvation, with a significant induction after 2 h (Supplemental Figure 11A). Regarding H3K27 trimethyltransferases, CLF was significantly induced after 3 days (Supplemental Figure 11B). Both expression patterns paralleled the timing of changes in histone marks observed at *IPT3*. Thus, we analyzed the effects of their mutations on *IPT3* chromatin profiles.

**Figure 4:**
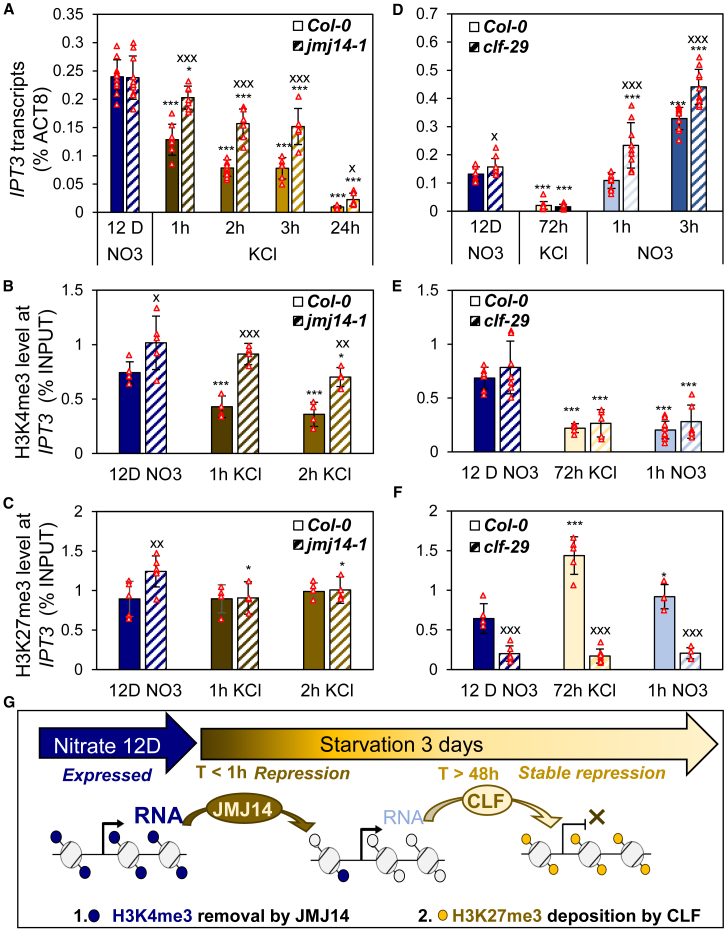
*IPT3* repression by nitrate starvation depends on H3K4me3 removal and coincides with H3K27me3 deposition. **(A–C)** Impact of *jmj14-1* mutant on *IPT3* transcripts **(A)** and H3K4me3 **(B)** or H3K27me3 **(C)** enrichment in the gene body region GB1. **(D–F)** Impact of *clf-29* mutant on *IPT3* transcripts **(D)** and H3K4me3 **(E)** or H3K27me3 **(F)** enrichment in GB1 (see also Supplemental Figure 12). The color code is based on time points illustrated in Figure 1A, with solid colors for Col-0 and striped colors for mutants. Data are presented as mean ± SD. *N* = 6–10 **(A)**, 4–6 **(B** and **C)**, 10–16 **(D)**, and 6–8 **(E** and **F)** biologically independent samples (red triangles). Asterisks denote statistically significant differences between 12 D NO_3_ and later time points (two-tailed Student’s *t*-test: ∗*p* < 0.05, ∗∗*p* < 0.01, and ∗∗∗*p* < 0.001). Differences between WT and mutants at each time point were tested similarly and are indicated by “X” (^x^*p* < 0.05). **(G)** Model of *IPT3* repression mediated by chromatin regulation. Early repression involves H3K4me3 removal by JMJ14 within a few hours, while H3K27me3 deposition by CLF occurs after 2 days of starvation at the *IPT3* locus.

The *jmj14-1* mutant exhibited higher *IPT3* expression than WT during starvation, correlating with increased H3K4me3 levels (Figures 4A and 4B). However, repression of *IPT3* still occurred. After 1 h of starvation, *jmj14-1* showed a significant reduction in *IPT3* transcript levels compared with nitrate-fed plants, although H3K4me3 levels remained unchanged (Figure 4B), indicating the involvement of additional repressor(s) independent of H3K4me3 demethylation. By 2 h, H3K4me3 levels were significantly reduced compared with the initial conditions, although they remained approximately twice as high as in WT plants. This suggests that another JMJ isoform may have a redundant role in H3K4me3 demethylation at *IPT3*. Under nitrate-supplied conditions, *jmj14-1* maintained higher H3K4me3 levels than WT, indicating active demethylation even in the presence of nitrate. This increase in H3K4me3 was also accompanied by elevated H3K27me3 levels (Figures 4B and 4C), likely contributing to transcript homeostasis and preventing overexpression, as previously proposed for *NRT2.1* (Bellegarde et al., 2018). After 24 h of starvation, *IPT3* transcript levels were significantly reduced in *jmj14-1*, although they remained higher than in the WT. Altogether, these findings identify JMJ14 as one of the H3K4me3 demethylases involved in the early repression of *IPT3*.

We next explored the role of H3K27me3 in maintaining the established repression of *IPT3* using the *clf-29* mutant, which lacks functional CLF. The *clf-29* mutant exhibited no increase in *IPT3* transcript levels after 72 h of starvation, despite a strong and widespread reduction of H3K27me3 at the *IPT3* locus (Figures 4D–4F). This finding indicates that CLF is the major methyltransferase responsible for H3K27me3 deposition at *IPT3*, but that loss of H3K27me3 alone is insufficient to reactivate *IPT3* expression during starvation. Interestingly, *clf-29* affected *IPT3* expression only under nitrate-sufficient conditions (see Figure 4D and discussion). Consistent with its role in maintaining stable gene repression, H3K27me3 also ensures proper spatiotemporal expression of its target genes. Because *IPT3* is specifically expressed in phloem companion cells, we confirmed that its spatial expression pattern remained unchanged in *clf-29* under nitrate supply (Supplemental Figures 12A and 12B). Chromatin immunoprecipitation (ChIP) further revealed that CLF binds to the *IPT3* locus predominantly under nitrate-starved (KCl) conditions (Supplemental Figure 12C), corresponding to the regions enriched for H3K27me3 (Figure 3C). These results suggest that CLF associates with the *IPT3* locus after 72 h of starvation, depositing H3K27me3 from the 5′ UTR through the gene body to stabilize *IPT3* repression in phloem companion cells.

Taken together, our findings reveal that early *IPT3* repression involves the removal of H3K4me3 by JMJ14, along with an additional unidentified repressor acting within the first few hours of starvation. Meanwhile, CLF-mediated H3K27me3 deposition secures stable repression of *IPT3* (Figure 4G).

### The two phases of *IPT3* induction by nitrate are controlled by H3K27me3 demethylation, independently of NLP6/NLP7, followed by H3K4me3 methylation

**Figure 5:**
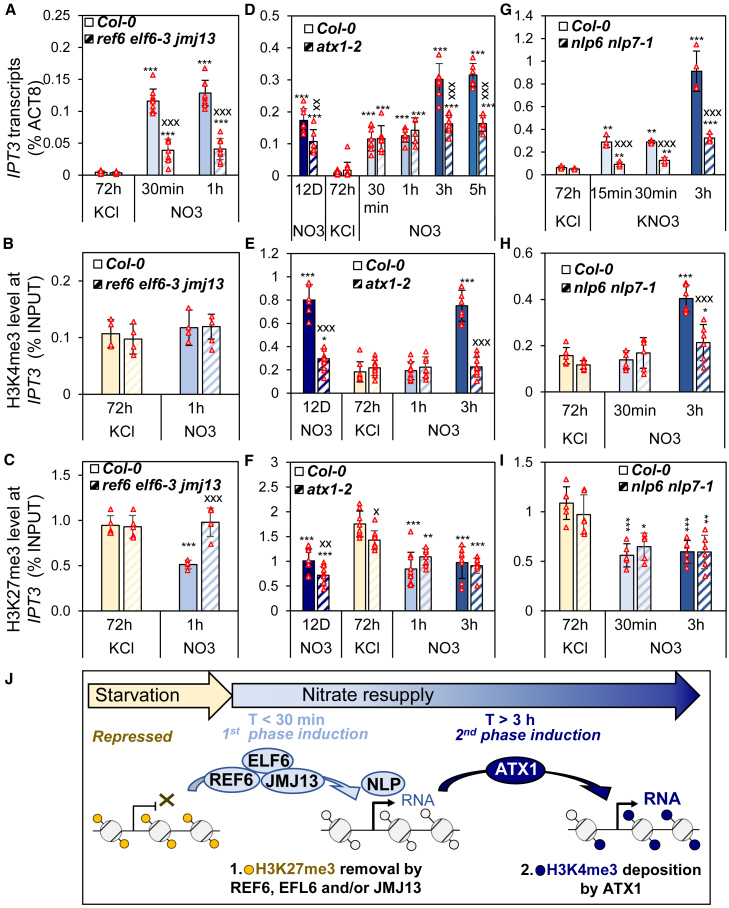
*IPT3* induction by nitrate depends on H3K27me3 removal and H3K4me3 deposition. **(A–C)** Impact of *ref6 elf6-3 jmj13* triple mutant on IPT3 transcript levels **(A)** and H3K4me3 **(B)** or H3K27me3 **(C)** enrichment in GB1 (see also Supplemental Figures 13 and 14). **(D–F)** Impact of *atx1-2* mutant on IPT3 transcript levels **(D)** and H3K4me3 **(E)** or H3K27me3 **(F)** enrichment in GB1. **(G–I)** Impact of *nlp6 nlp7-1* double mutant on IPT3 transcript levels **(G)** and H3K4me3 **(H)** or H3K27me3 **(I)** enrichment in GB1. The color code is based on time points illustrated in Figure 1A, with solid colors for Col-0 and striped colors for mutants. Data are presented as mean ± SD. *N* = 8–10 **(A)**, 4 **(B** and **C)**, 6–10 **(D)**, 8–13 **(E** and **F)**, 4 **(G)**, and 6 **(H** and **I)** biologically independent samples (red triangles). Asterisks denote statistically significant differences between KCl and later time points (two-tailed Student’s *t*-test: ∗*p* < 0.05, ∗∗*p* < 0.01, and ∗∗∗*p* < 0.001). Differences between WT and mutants within each time point were tested similarly and are indicated by “X” (^x^*p* < 0.05). **(J)** Model of *IPT3* induction mediated by chromatin regulation. Early induction involves H3K27me3 removal by REF6, ELF6, or JMJ13 within a few minutes, while H3K4me3 deposition by ATX1 after 3 h is necessary for the second phase of *IPT3* induction. NLP6 or NLP7 is not required for H3K27me3 removal but remains important for proper *IPT3* induction and H3K4me3 enrichment.

Next, we examined *IPT3* induction after nitrate resupply following 3 days of starvation. We hypothesized that an early nitrate response, mediated by transcription factors such as NLP7, initiates *IPT3* induction by recruiting H3K27me3 demethylases to remove the repressive H3K27me3 mark, leading to a weak *IPT3* induction. This could be followed by a second, stronger induction after 3 h, potentially mediated by H3K4 tri-methylation through H3K4 methyltransferase. To identify chromatin effectors involved in this process, we analyzed the expression profiles of H3K27me3 demethylases and H3K4 methyltransferases during N starvation (Supplemental Figure 13). Among the H3K27me3 demethylases, *REF6* and *JMJ30* were induced by nitrate within 15 min and 3 h, respectively, with *REF6* showing a stronger and faster response (Supplemental Figure 13A). Among the ATX and ATXR families of H3K4 methyltransferases, ATX1 was the only member induced by nitrate within 3 h (Supplemental Figure 13B). Both REF6 and ATX1 were induced at similar times to the chromatin changes observed at *IPT3*. Thus, we analyzed the effects of their mutations. Given that the H3K27me3 demethylases REF6, ELF6, and JMJ13 are functionally redundant paralogs (Yan et al., 2018; Cheng et al., 2020), we used the *ref6 elf6-3 jmj13* triple mutant to explore the role of REF6 in *IPT3* induction by nitrate (Yan et al., 2018) (Figures 5A–5C). In this mutant, nitrate-induced expression of *IPT3* was highly variable: in two independent experiments, no induction was observed (Supplemental Figure 14A), whereas in three other experiments, a weak induction occurred (Figure 5A). When induced, *IPT3* transcript levels in the mutant reached only about ⅓ of those in WT plants, even after 1 h of nitrate resupply (Figure 5A). This reduced induction was not due to impaired nitrate signaling, as nitrate sentinel genes were normally induced in the triple mutant (Supplemental Figures 14B–14E). H3K4me3 levels in the triple mutant were similar to those in WT (Figure 5B). Consistent with the role of H3K27me3 demethylases, H3K27me3 levels in the *ref6 elf6-3 jmj13* mutant remained unchanged between starvation and 1 h of nitrate resupply, whereas in the WT, H3K27me3 decreased following nitrate resupply (Figure 5C). Altogether, these results demonstrate that REF6, ELF6, and/or JMJ13 are key effectors of H3K27me3 demethylation at *IPT3*, playing an important role in early nitrate-mediated induction.

We then examined the role of H3K4me3 in the second phase of *IPT3* induction following nitrate repletion. The *atx1-2* mutant showed a distinctive phenotype (5D-F). After 12 days, *IPT3* expression in the mutant was reduced by half, correlating with a strong reduction in H3K4me3 enrichment and a slight decrease in H3K27me3. H3K4me3 enrichment at 12 days was significantly higher than at other time points in *atx1-2*, suggesting that in *atx1-2*, the modest increase in H3K4me3, together with a decrease in basal H3K27me3 levels, was sufficient to sustain *IPT3* expression under constant nitrate compared with other time points. These observations indicate that (i) ATX1 is the predominant effector of H3K4me3 deposition at *IPT3*, although a secondary effector may partially compensate under constant nitrate conditions, and (ii) the balance between H3K4me3 and H3K27me3 is crucial for proper transcriptional regulation of *IPT3*. Interestingly, the initial phase of *IPT3* induction upon nitrate resupply occurred normally in *atx1-2*, whereas the second phase of induction was completely abolished, even after 5 h of nitrate resupply. These results demonstrate that H3K4 trimethylation by ATX1 is required for the second phase of *IPT3* induction.

NLP7 is the only transcription factor previously proposed to act as an effector of nitrate-mediated *IPT3* induction (Abualia et al., 2022)*.* Surprisingly, analysis of the *nlp6 nlp7-1* double mutant revealed that, despite a strong reduction in *IPT3* induction, H3K27me3 demethylation occurred normally within 30 min (5G-I). H3K4me3 enrichment was also detected after 3 h of nitrate resupply, though at reduced levels compared with WT. These results suggest that NLP6 and/or NLP7 act downstream of H3K27me3 removal and upstream of H3K4me3 deposition to ensure proper *IPT3* regulation.

Together, our results suggest that the early phase of *IPT3* induction after nitrate resupply requires reactivation of the locus via H3K27me3 removal by REF6, ELF6, and/or JMJ13 demethylases, followed by activation through NLP6/NLP7. The subsequent stronger increase in *IPT3* transcript levels after 3 h of nitrate repletion depends on H3K4me3 deposition mediated by ATX1 (Figure 5G).

### The dynamics of H3K4me3 and H3K27me3 are crucial for CK production

**Figure 6:**
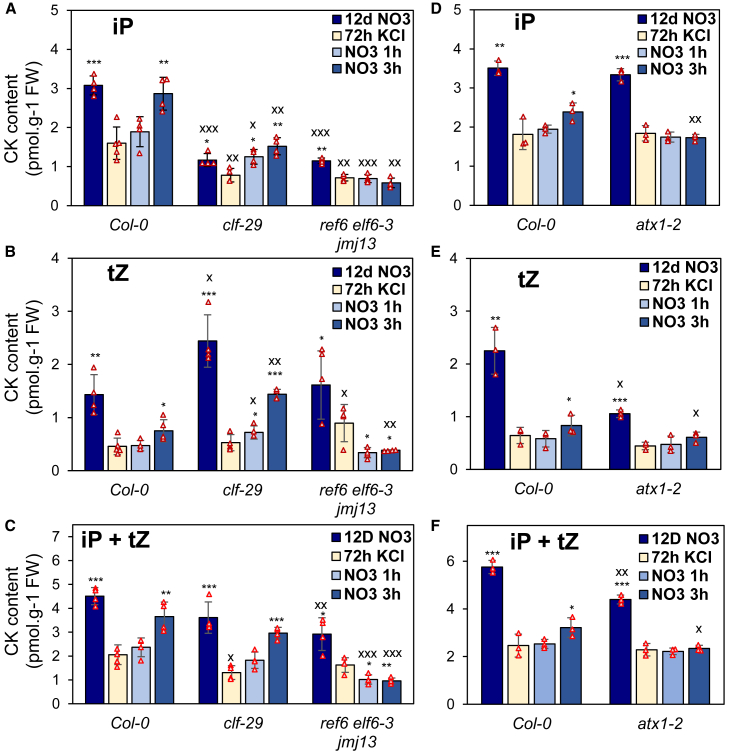
Chromatin regulation is required for proper root CK output during nitrate fluctuation. Root CK profiles during nitrate fluctuation in H3K27-related mutants *clf-29* and the *ref6 elf6-3 jmj13* triple mutant **(A–C)** or in *atx1-2***(D–F)** (see also Supplemental Figure 15). Panels in **(C)** and **(F)** show the sum of iP and tZ for each sample. Data are presented as mean ± SD. *N* = 4–5 **(A–C) and** 3 **(D–F)** biologically independent samples (red triangles). Asterisks denote statistically significant differences between KCl and other time points (two-tailed Student’s *t*-test: ∗*p* < 0.05, ∗∗*p* < 0.01, and ∗∗∗*p* < 0.001). Differences between WT and mutants at each time point were performed similarly and are indicated by “X” (^x^*p* < 0.05).

To assess the physiological impact of histone modifications on CK biosynthesis, we quantified root CK levels in H3K27me3-related mutants (6A-C, Supplemental Figures 15A-D) and in the *atx1-2* mutant (6D-E, Supplemental Figures 15E-H). WT plants showed CK profiles consistent with those described earlier (Figure 6, Figure 1).

Interestingly, in the *clf-29* mutant, which exhibited elevated *IPT3* expression under nitrate supply, we observed lower levels of iP-type CKs than in the WT, accompanied by an increase in tZ-type CKs only under nitrate conditions (6A-B). Moreover, tZ levels in *clf-29* increased significantly after 1 h of nitrate resupply and reached WT levels after 3 h. These results suggest that the absence of H3K27me3 deposition in *clf-29* accelerates the conversion of iP to tZ under nitrate-supplied conditions. To test this hypothesis, we summed iP and tZ levels obtained in each sample for statistical analysis (Figure 6C). Except for a significant reduction under KCl treatment, total CK content (iP + tZ) in *clf-29* did not differ significantly from that of the WT, supporting the conclusion that faster iP-to-tZ conversion occurs in *clf-29*.

In the *ref6 elf6-3 jmj13* triple mutant, the most prominent phenotype was observed upon nitrate resupply, when both iP- and tZ-type CKs showed a general decrease beginning at 1 h. This aligns with the lack of *IPT3* transcriptional activation in the triple mutant.

The effect of the *atx1-2* mutation on CK biosynthesis was examined separately (6D-F, Supplemental Figure 13E-H). While no decrease in iP was observed under constant nitrate conditions, tZ-type CKs were markedly decreased. CK levels also remained relatively stable between 1 h and 3 h of nitrate resupply in *atx1-2*, especially for the iP type, consistent with the loss of the second phase of *IPT3* induction. When the iP and tZ levels were summed, the overall level of active CKs was reduced at the same time points as IPT3 expression—under constant nitrate and after 3 h of resupply (Figure 6F).

These results demonstrate that chromatin-mediated transcriptional regulation of *IPT3* is an important factor controlling root CK biosynthesis in response to fluctuating nitrate availability.

## Discussion

**Figure 7:**
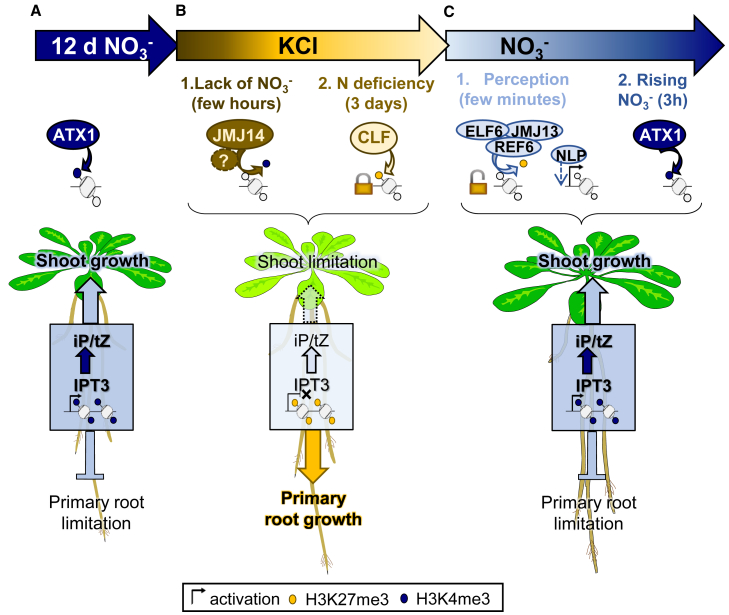
Model of chromatin-mediated CK biosynthesis regulation during fluctuating nitrate and its impact on growth. **(A)** Under constant nitrate conditions, nitrate stimulates *IPT3* expression through H3K4 trimethylation mediated by ATX1, leading to the biosynthesis of iP and tZ that locally limits primary root growth and systemically stimulates shoot growth (via long-distance nitrate signaling). **(B)** In the absence of nitrate, CK downregulation is rapidly triggered by an unknown repressor, leading to JMJ14-mediated H3K4me3 demethylation at *IPT3*. Lower CK levels relieve nitrate-mediated repression of primary root growth, promoting elongation while suppressing shoot growth. After 3 days of starvation, a global stress response induced by deficiency locks CK biosynthesis genes via CLF-mediated H3K27 trimethylation. **(C)** Nitrate perception rapidly unlocks CK biosynthesis genes through H3K27me3 demethylation mediated by REF6, ELF6, or JMJ13, followed by NLP-dependent induction (direct or indirect). Initially, iP-type CKs are produced. As nitrate content rises, ATX1-mediated H3K4 trimethylation enhances *IPT3* expression, thereby activating *CYP735A2* and *ABCG14*, which promote tZ biosynthesis and transport to stimulate shoot growth while restricting root growth.

In this study, we unraveled a specific function of IPT3 in plant acclimation to fluctuating nitrate environments and identified the molecular mechanisms underlying its regulation, which relies on the balance between H3K4me3 and H3K27me3 enrichment. By organizing our results over time, we built a model illustrating how chromatin-mediated regulation of root CK biosynthesis enables growth acclimation to nitrate fluctuations and how histone modifications contribute to this process (Figure 7).

Under constant nitrate conditions (Figure 7A), the methyltransferase ATX1 mediates H3K4me3 deposition at *IPT3*, leading to CK biosynthesis in roots. The CKs produced through *IPT3* regulation, including tZ-type CKs, help maintain the root/shoot growth balance by restricting primary root development to support proper shoot growth. In the *atx1-2* mutant, no significant decrease in iP content was observed at 12 days (Figure 6), suggesting that residual *IPT3* expression in *atx1-2* was sufficient to sustain iP-type CK biosynthesis and/or was compensated by IPT5 or IPT7 to maintain iP homeostasis under stable nitrate supply. Unexpectedly, REF6, ELF6, and/or JMJ13 could also be involved in iP homeostasis under constant nitrate, as a reduction in CK, especially iP, occurred in the triple mutant (Figure 6) despite normal *IPT3* and *CYP735A2* expression before starvation (Supplemental Figure 16). This suggests that CK downregulation in the triple mutant is not due to impaired CK biosynthesis but may instead result from increased CK degradation. Consistent with the *atx1-2* interpretation and based on the *ipt3-2* and *iptTM* phenotypes, we also observed partial or localized complementation by IPT5 or IPT7 for CK production in nitrate-grown *ipt3-2* plants. However, loss of *IPT3* (*ipt3-2* and *iptTM*) disrupted the root-shoot growth balance, leading to a starvation-like phenotype even under nitrate conditions, which was rescued by reintroduction of *IPT3* in the triple mutant (IPT3-RFP line), confirming the dominant role of IPT3. Nevertheless, IPT5 and/or IPT7 also contribute to developmental responses and may have specific functions. CK has been reported to be important for lateral root positioning (Chang et al., 2015). Although no significant differences were observed between WT and *ipt3-2* (Supplemental Figure 6F), *iptTM* had an increased number of lateral roots, a phenotype not rescued in IPT3-RFP, suggesting that IPT5 or IPT7 are more important for lateral root development. In particular, IPT5, whose promoter is active in primordia and lateral roots, may act differently from *IPT3*, whose promoter activity under nitrate was first detected at 1 dpg in primary roots and root tips (Supplemental Figure 17A). *IPT3* expression at lateral root tips may suggest a more general function in restricting root elongation under nitrate supply. Given the *IPT3* expression profile at the main root tip (Supplemental 17A-B) and its effect on root meristem size (Supplemental Figure 8), we observed a weak but distinct IPT3-RFP signal in root tips. Although the signal was more diffuse across all tissues than in upper regions (Supplemental Figure 3), the RFP signal correlated with GFP expression just before the transition zone (Supplemental Figures 17B and 17C). This signal decreased during starvation (Supplemental Figure 17C), suggesting that it is a true signal rather than autofluorescence. Further analysis of the meristem signal using a fluorescent protein other than RFP should be performed to reduce autofluorescence artifacts. Based on these results, we propose that, in addition to its function in phloem companion cells along the root, IPT3 may also act locally near the transition zone to control meristematic cell differentiation and inhibit primary root growth. Although we did not directly examine IPT3 function and profile in lateral root tips, our results reveal that under nitrate-sufficient conditions, IPT3 functions as a key regulator of global CK output, thereby limiting primary root growth.

During nitrate starvation (Figure 7B), the rapid decrease in root nitrate content is concomitant with active repression of CK biosynthesis genes. In the absence of *de novo* CK biosynthesis, CK content decreases (1C-D), especially for tZ-type CKs (factor 3), probably due to its role in long-distance nitrate signaling, which must be suppressed during starvation. Both tZ-type CKs and active iP reached basal levels within 1 day of starvation, leading to an increase in primary root growth rate. This rapid downregulation suggests that, in addition to transcriptional repression, an additional layer of control such as CK degradation may contribute to the observed decline in CK levels. IPT3 repression occurs in two steps: initially through an unknown repressor, probably a transcription factor, followed by JMJ14-mediated H3K4me3 demethylation that inactivates *IPT3* expression within a few hours. As noted in the results, other JMJ family members could act redundantly at the *IPT3* locus. Among the closest homologs of JMJ14, JMJ15 and JMJ18 have been shown to act redundantly with JMJ14 under high-temperature stress (Cui et al., 2021) and are expressed in the root vasculature along with *JMJ14* (Cattaneo et al., 2019), *IPT3*, and *CYP735A2* (Sakakibara, 2021). Although neither JMJ15 nor JMJ18 was induced by starvation (Supplemental Figure 11), they constitute good candidates for JMJ14 compensation. The nature of the preliminary repressor remains unknown, but because the reduction in *IPT3* transcript levels is greater than that in H3K4me3 enrichment (factors of 3 and 2, respectively), an additional layer of regulation is likely involved. It has been shown that transcriptional repressors such as TOPLESS can recruit histone modifiers, especially histone deacetylases, to repress gene expression and regulate multiple plant developmental processes often associated with phytohormone signaling (Plant et al., 2021). Similarly, ABA biosynthesis repression during seed germination involves the rapid removal of H3K4me3 and H3Kac mediated by an RNA-binding protein within a few hours, followed by H3K27me3 deposition after 48 h to stabilize repression (Yang et al., 2022)—a process similar to our observation of *IPT3* repression under nitrate starvation. Indeed, after 3 days of starvation, CLF-mediated H3K27me3 enrichment occurs at *IPT3*. This coincides with whole-plant nitrate deficiency, as revealed by the onset of chlorophyll degradation (Figure 1, Supplemental Figure 1), suggesting that deficiency-induced stress responses lead to chromatin locking of the *IPT3* locus and thereby repress iP- and tZ-type CK biosynthesis. For many other stress responses, CK biosynthesis or signaling is often repressed, and reduced CK levels promote stress tolerance (Cortleven et al., 2019). Thus, plants must balance their developmental program between CK-mediated growth promotion and activation of stress responses. Interestingly, perturbation of H3K27me3 dynamics had no effect during starvation. Several studies have reported that the loss of H3K27me3 is not systematically associated with increased gene expression (Aichinger et al., 2011; Bouyer et al., 2011; Farrona et al., 2011). Indeed, H3K27me3 mainly maintains repression rather than establishing it. The NLP7 protein localizes to the nucleus only in the presence of nitrate, while it is actively excluded in the absence of nitrate (Marchive et al., 2013; Liu et al., 2017). Therefore, the transcription factor required for *IPT3* induction (not necessarily NLP7) may not be expressed or present in the nucleus during starvation. Supporting this hypothesis, *IPT3* expression under nitrate-sufficient conditions is consistently higher in *clf-29* than in WT, indicating that H3K27me3 influences nitrate-dependent transcription and is mainly deposited under KCl conditions (Figure 4, Supplemental Figure 12). However, the altered CK content in *clf-29* (Figure 6), characterized by a faster conversion of iP to tZ under nitrate conditions, suggests that deficiency-induced H3K27me3 enrichment functions to delay CK production by locking the *IPT3* locus, thereby modulating nitrate satiety feedback after extended starvation. In rice, another member of the PRC2 complex (OsEMF2) regulates CK biosynthesis genes (*OsIPT2* and *OsLOG1*) to control the cellularization of the endosperm (Cheng et al., 2021). In *Arabidopsis*, it has been proposed recently that the transcriptional repressor KNUCKLES regulates auxin transport and cytokinin biosynthesis via H3K27me3 deposition at *PIN1* and *IPT7*, respectively, to control floral meristem determinacy (Wang et al., 2024). These observations suggest that PRC2 and H3K27me3 may play a broader role in CK biosynthesis by regulating different physiological functions of CK. A similar relationship between increased *IPT3* expression (and CK production) and decreased H3K27me3 levels at *IPT3* has also been observed in *sdg26* mutant, impaired in the H3K36 methyltransferase SDG26 (Liu et al., 2016), during trichome development (Zeng et al., 2023). Lastly, a starvation-specific phenotype was observed in the triple mutant for H3K27 demethylases, corresponding to decreased iP and tZRP levels and increased tZ during starvation (6A-B, Supplemental Figures 15A-D). This phenotype also correlated with stronger *CYP735A2* expression after 3 days of starvation in the triple mutant (Supplemental Figure 17A). These findings suggest that REF6, ELF6, and/or JMJ13 may participate in the activation of a *CYP735A2*-specific repressor under starvation conditions.

Nitrate replenishment responses also occur in two steps (Figure 7C). Early nitrate perception leads to a rapid *IPT3* induction and correlates with a marked decrease in H3K27me3, thereby reactivating the locus (unlocking). H3K27me3 demethylation mediated by REF6, ELF6, and/or JMJ13 appears necessary for *IPT3* induction. The strong variability observed in the triple mutant background suggests the existence of additional regulatory mechanisms that may compensate for the lack of H3K27me3 demethylation. It has been shown that H3K27me3 can be removed through H3 eviction under cold stress (Kwon et al., 2009). However, we did not observe a decrease in H3 levels in the triple mutant (Supplemental Figure 18). *IPT3* is also targeted by the ATP-dependent chromatin-remodeling complex SWI/SNF (Jégu et al., 2015), which can remove, replace, or reposition nucleosomes, thereby enabling transcriptional machinery access to target genes (Diego-Martin et al., 2022). To clarify these interactions, the specific contributions of individual JMJ mutants, the SWI/SNF complex, and nucleosome density should be further examined. The second phase of the nitrate response, occurring after 3 h of resupply, corresponds to a significant increase in root nitrate content and a second increase in *IPT3* transcript levels, which depends on H3K4 trimethylation by ATX1. Consistent with this, in *atx1-2*, the absence of *IPT3* induction during the second phase reduces *CYP735A2* expression (Supplemental Figure 19A) and prevents the production of tZR and tZ (6E, 6D, Supplemental Figures 15E-H). Although we cannot exclude the possibility that the produced tZR is translocated to the shoot, the expression of *ABCG14*, which is induced by CK in an IPT3-dependent manner (Supplemental Figures 2I-J), is also reduced after 3 h of nitrate replenishment (Supplemental Figure 19B). This reinforces the notion that stronger *IPT3* induction and CK accumulation drive a second wave of *CYP735A2* and *ABCG14* induction, independently of nitrate increase (Supplemental Figures 2G-J and Supplemental Figure 4). This likely constitutes the CK-mediated secondary nitrate response, marking the onset of the nitrate satiety signal and promoting shoot growth while limiting root growth. This interpretation is further supported by Supplemental Figure 1, which shows that after 3 h of nitrate resupply, chlorophyll content is restored and *NLP7* expression is reduced, suggesting that the increase in root nitrate content results from nitrate storage in root cell vacuoles or reduced nitrate translocation/assimilation. Such a two-step induction of *IPT3* permits precise control of *de novo* CK biosynthesis and rapid root growth acclimation to nitrate resupply, a process that is severely compromised in the *ipt3-2* mutant. The *ipt3-2* phenotype does not appear to result from defective nitrate sensing. Indeed, the two known nitrate sensor and sentinel genes, *NRT1.1* (Ho et al., 2009) and *NLP7* (Liu et al., 2022), responded normally to nitrate fluctuations in the *ipt3-2* mutant (Supplemental Figure 20). Notably, *NRT1.1* induction was even stronger in *ipt3-2* than in WT upon nitrate resupply, consistent with the repressive effect of CK on this gene (Kiba et al., 2011). Altogether, chromatin-mediated transcriptional regulation of *IPT3* under fluctuating nitrate conditions emerges as a key determinant of CK output and growth acclimation.

How nitrate fluctuations recruit different chromatin effectors at CK biosynthesis gene loci remains to be elucidated and likely involves transcription factors. Among the well-characterized transcription factors implicated in nitrate nutrition, there is, to our knowledge, no evidence of their direct association with chromatin effectors for transcriptional regulation. The rapid induction of *IPT3* (Supplemental Figure 2), similar to that of nitrate sentinel genes, strongly suggests that the primary nitrate response mediated by NLP7 could be a key determinant of *IPT3* activation. However, analysis of the *nlp6 nlp7-1* double mutant revealed that H3K27me3 removal occurs normally despite a weaker *IPT3* induction than in WT. This indicates that another transcription factor may be involved, but our data suggest that at least the removal of H3K27me3 could be a prerequisite for NLP function in *IPT3* expression. We cannot exclude the possibility that NLP7 regulates *IPT3* expression indirectly through another transcription factor. Indeed, analyses of available *in planta* ChIP-chip data (Marchive et al., 2013) and root cell protoplast ChIP-seq data (Alvarez et al., 2020) did not identify *IPT3* as a direct target of NLP7, suggesting an indirect effect or that the observed phenotype may result from the loss of NLP6 rather than NLP7. The precise roles of NLP7 and NLP6 in *IPT3* regulation thus remain to be clarified, but they appear to contribute only marginally, if at all, to chromatin changes at *IPT3*.

This study provides an integrated view of plant acclimation to nitrate fluctuations, including its temporal dynamics and molecular mechanisms. We identified several chromatin effectors that warrant further analysis, especially regarding their interactions with transcription factors. Our study reveals an additional layer of regulation connecting nitrate nutrition and hormonal signaling in the control of plant growth. This work offers new perspectives for understanding the complex interplay among chromatin, hormonal, and nutritional regulation underlying plant acclimation to fluctuating nutrient conditions. Such understanding could ultimately inform new strategies for crop improvement.

## Methods

### Plant material

The *Arabidopsis thaliana* accession used in this study was *Col-0*. The mutant alleles and transgenic lines included *clf-29* (De Lucas et al., 2016), *ProCLF:CFP:CLF;clf-29* (De Lucas et al., 2016), *elf6*-*3 ref6*^*C*^ *jmj13*^*G*^ (Yan et al., 2018), *atx1-2* (Pien et al., 2008), *jmj14-1* (Searle et al., 2010), *nlp6 nlp7-1* (Maeda et al., 2018), and the *ipt3-2* or *ipt3 ipt5 ipt7* triple mutant (Miyawaki et al., 2006), all of which have been previously characterized. The generation of the IPT3-RFP line is described below.

### Plasmid construction and generation of IPT3-RFP transgenic lines

Fragments corresponding to RFP and the genomic region of the *IPT3* locus—from the distal promoter (−1959) through the coding region but excluding the stop codon—were amplified using PrimeSTAR GXL DNA polymerase (Takara) with specific primers (listed in Supplemental Table 1). The PCR products were cloned into the pENTR3C vector at the SalI/EcoRV restriction sites using NEBuilder, sequenced, and transferred into the Gateway binary vector pBA002a-GW, a derivative of pBA002a (Kiba et al., 2018), with the GATEWAY LR Clonase II enzyme mix (Invitrogen). The construct was introduced into *Agrobacterium tumefaciens* strain EHA105, and *iptTM* plants were transformed by the floral dip method. Transformants were selected on MS medium containing 1% sucrose and 10 mg l^−1^ bialaphos sodium salt. The root recovery phenotype was used to identify three representative lines. Only one of these representative lines was used for further characterization.

### Growth conditions

Most experiments were performed using roots from 12- to 15-day-old seedlings grown hydroponically. Root phenotyping (Figures 2F–2H) was performed on seedlings grown on Petri dishes. In both systems, plants were grown at 22°C under long-day conditions (16 h light and 8 h dark) in medium containing 750 μM MgSO_4_, 625 μM KH_2_PO_4_, 1500 μM CaCl_2_, 75 μM Fe(II)-EDTA, and micronutrients (55 nM CoCl_2_, 53 nM CuCl_2_, 50 μM H_3_BO_3_, 2.5 μM KI, 50 μM MnCl, 0.52 μM Na_2_MoO_4_, and 15 μM ZnCl_2_). MES (0.5 g.l^−1^) was added to buffer the medium, and the PH was adjusted to 5.8. Agar (0.7%) was then added to solidify the culture medium in Petri dishes. Depending on the treatment, hydroponic solutions were supplemented with either 1 mM KNO_3_ or 1 mM KCl (Figure 1A). To maintain pH stability and nutrient concentration during the first 12 days of hydroponic culture, the nitrate solution was renewed every 3–-4 days and again 24 h before either harvest or initiation of KCl treatment at day 11.

### Phenotyping

To investigate the impact of *ipt3-2*, *iptTM*, and IPT3-RFP on growth and to ensure synchronized germination, seeds were transferred with a toothpick to new Petri dishes (1 mM nitrate) as soon as the radicle was visible (1–2 mm). This point was designated as day 0 (germination). Each day (24 h ± 10 min), the root tip was marked (visible in Supplemental Figure 6A), and root length was measured from scanned images using ImageJ software (segmented line tool). Lateral root numbers were counted manually with the aid of a magnifier before transfer at 12 dpg. Root growth rate was calculated as the difference in primary root length between time points, expressed as the length (cm) produced per day. Media were renewed once, at 7 dpg, by transferring plants to fresh Petri dishes containing nitrate. During this process, the number of samples (initially about 150 plants per genotype) decreased due to harvesting subsamples for root and shoot FW measurements (Sartorius ME5 microbalance) and root meristem observations (see supplemental methods).

### CK extraction and quantification

CKs were extracted and determined from approximately 20 mg (FW) of root tissues, as described previously (Kojima et al., 2009), using ultra-performance liquid chromatography–tandem mass spectrometry (ACQUITY UPLC System/XEVO-TQ-XS; Waters) equipped with an ACQUITY UPLC HSS T3 C_18_ column (1.8 (μm, 2.1 × 100 mm; Waters).

### NO_3_ content

NO_3_ was extracted and determined from about 10 mg of roots or 20 mg (FW) of shoots as previously described (Hachiya and Okamoto, 2017), with minor modifications. Roots from the KNO_3_ solution were rinsed for 5 s in KCl solution to remove adhering nitrate, blotted dry, weighed, and frozen in liquid nitrogen. Boiling Milli-Q water was added to frozen samples to prevent NR activity and incubated for 20 min at 100°C with shaking (700 rpm). The assay was performed by adding 10 μl of extract to 40 μl of either 5% (w/v) salicylic acid-sulfuric acid solution or sulfuric acid alone (used to estimate background noise). After 20 min, the reaction was stopped by adding 1 ml of 8% NaOH. A standard curve was prepared using serial dilutions of KNO_3_ (0, 0.125, 0.25, 0.5, 1, 2, 4, 6, and 8 mM). Absorbance at 410 nm (OD_410_) was measured in a microplate spectrophotometer. Nitrate concentrations were calculated from the standard curve, and final values were obtained by subtracting the corresponding background.

### RNA extraction and expression analysis

Root samples were frozen in liquid nitrogen, and total RNA was extracted using TRIzol reagent (Invitrogen). RNA samples were treated with DNase I (RQ1 Promega), quantified, and adjusted prior to reverse transcription with ReverTra Ace qPCR RT Master Mix (TOYOBO). Transcript abundance was determined by RT–qPCR using a SYBR Kit (Takara) following the manufacturer’s instructions. Gene expression levels were normalized to *ACT8* as an internal reference. Primer sequences used for qPCR are listed in Supplemental Table 2.

### ChIP experiments

ChIP assays were performed to quantify histone modification enrichment as previously described (Bellegarde et al., 2018), with minor modifications. Nuclei were isolated in nuclei isolation buffer (20 mM PIPES–KOH [pH 7.6], 1 M hexylene glycol, 10 mM MgCl2, 0.1 mM EGTA, 15 mM NaCl, 60 mM KCl, 0.5% Triton X-100, 5 mM β-mercaptoethanol, and protease inhibitor cocktail [Complete Tablets EASYpack, Roche]) and resuspended in nuclei lysis buffer. Chromatin was immunoprecipitated with antibodies against H3K27me3 (4 μg, Millipore 07-449) and H3K4me3 (2 μg, Diagenode C15410030). Immunoprecipitated DNA was purified by phenol-chloroform extraction and quantified by qPCR.

ChIP data were normalized to input DNA (10% of total sample, adjusted to 100%). Enrichment was calculated as the percentage of input using the formula: Enrichment (%) = (2^−(Cp IP – Cp Input adj)^ × 100). Primer sequences used for ChIP-qPCR are listed in Supplemental Table 1.

### Data analysis and presentation

All numerical values are expressed as mean ± SD, based on at least three biologically independent replicates. Except for plant growth measurements, each sample represented a pool of 8–16 plants, depending on the purpose of the experiment and material availability. Comparable quantities were harvested at each time point within a given experiment. All figures presented in the main text were reproduced in at least two independent experiments to ensure robustness. Statistical significance of treatment effects was assessed by one-way ANOVA followed by Tukey's HSD post-hoc test (*p* < 0.05) using Jamovi software. Pairwise comparisons between WT and transgenic lines were conducted using a two-tailed Student’s *t*-test in Microsoft Excel. Significance thresholds were defined as follows: ∗*p* < 0.05, ∗∗*p* < 0.01, ∗∗∗*p* < 0.001.

## Funding

This research was supported by the Programs for Promoting the Enhancement of Research Universities, 10.13039/501100004823Nagoya University (FY2019-2022); a KAKENHI Grant-in-Aid for Scientific Research on Innovative Areas (no. JP17H06473S) from the 10.13039/501100001700Ministry of Education, Culture, Sports, Science and Technology; 10.13039/501100001691JSPS KAKENHI Grants-in-Aid for Scientific Research (A) (nos. JP19H00931 and JP23H00324); a 10.13039/501100001691JSPS KAKENHI Grant-in-Aid for Early-Career Scientists (no. 2824K18138); and the Tokai Pathways to Global Excellence (T-GEx) program, part of the MEXT Strategic Professional Development Program for Young Researchers (no. 0121an0002).

## Acknowledgments

We thank Dr. Antoine Martin for providing chromatin mutant seeds and Dr. Andres Maturana for serving as an external reviewer of the manuscript. We also thank Clarissa Frances Frederica (student of Dr. Louis J. Irving) and Dr. Shigeki Wada (Shimoda Marine Research Center) for their assistance with the MS run and system access, respectively. No conflict of interest declared.

## Author contributions

Conceptualization, F.B. (lead), H.S. (support), and T.K. (support); writing – original draft, F.B. (lead), H.S. (support), T.K. (support), and L.J.I. (support); writing – review & editing, all authors; design and data analysis, F.B. (lead) and T.K. (support); most experiments, F.B. (lead), O.T. (support), and M.S.; CK experiment, F.B. (lead), M.Y.-K. (lead), M. Shibutani (support), and K.M. (support); influx experiment, M. Kuriyama, F.B., and L.J.I.; line construction, T.K.; funding acquisition, H.S. and F.B.

## Supplemental information

Document S1. Supplemental Figures 1–20, Supplemental Tables 1 and 2, and supplemental methods

Document S2. Article plus supplemental information
