# Supplementary material for: Fluctuations in nitrate availability impact cytokinin biosynthesis through histone modifications of IPT3 in Arabidopsis roots for growth acclimation
Source: Plant Commun. 2025 Sep 23;6(11):101531. doi: 10.1016/j.xplc.2025.101531 (PMC12785166; doi:10.1016/j.xplc.2025.101531)
Supplement: Document S1. Supplemental Figures 1–20, Supplemental Tables 1 and 2, and supplemental methods [file mmc1.pdf]

Plant Communications, Volume *6*

Supplemental information

Fluctuations in nitrate availability impact cytokinin biosynthesis through histone modifications of *IPT3* in *Arabidopsis* roots for growth acclimation

Fanny Bellegarde, Olivia Tjahjono, Mika Yoshino-Kida, Takatoshi Kiba, Miki Shibutani, Mei Kuriyama, Louis J. Irving, Mikiko Kojima, Kazuki Miyata, and Hitoshi Sakakibara

Document S1

Supplemental Figures, Tables, methods, and references for:

Fluctuation in nitrate availability impacts cytokinin biosynthesis through histone modifications of *IPT3* in Arabidopsis roots for growth acclimation

Fanny Bellegarde[1,2*], Olivia Tjahjono[1], Mika Yoshino-Kida[1], Takatoshi Kiba[1a], Miki Shibutani[1], Mei Kuriyama[1], Louis J. Irving[3], Mikiko Kojima[4], Kazuki Miyata[1] and Hitoshi Sakakibara[1,4]

[1] Graduate School of Bioagricultural Sciences, Nagoya University, Aichi 464-8601, Japan

[2] Institute for Advanced Research, Nagoya University, Aichi 464-8601, Japan

[3] Institute of Life and Environmental Sciences, University of Tsukuba, Tennodai 1-1-1, Tsukuba 305-8577, Japan

[4]RIKEN Center for Sustainable Resource Science, Tsurumi, Yokohama 230-0045, Japan.

[a] Present address: Institute of Plant Science and Resources, Okayama University, 2-20-1, Chuo, Kurashiki, Okayama 710-0046, JAPAN

Supplemental Figures

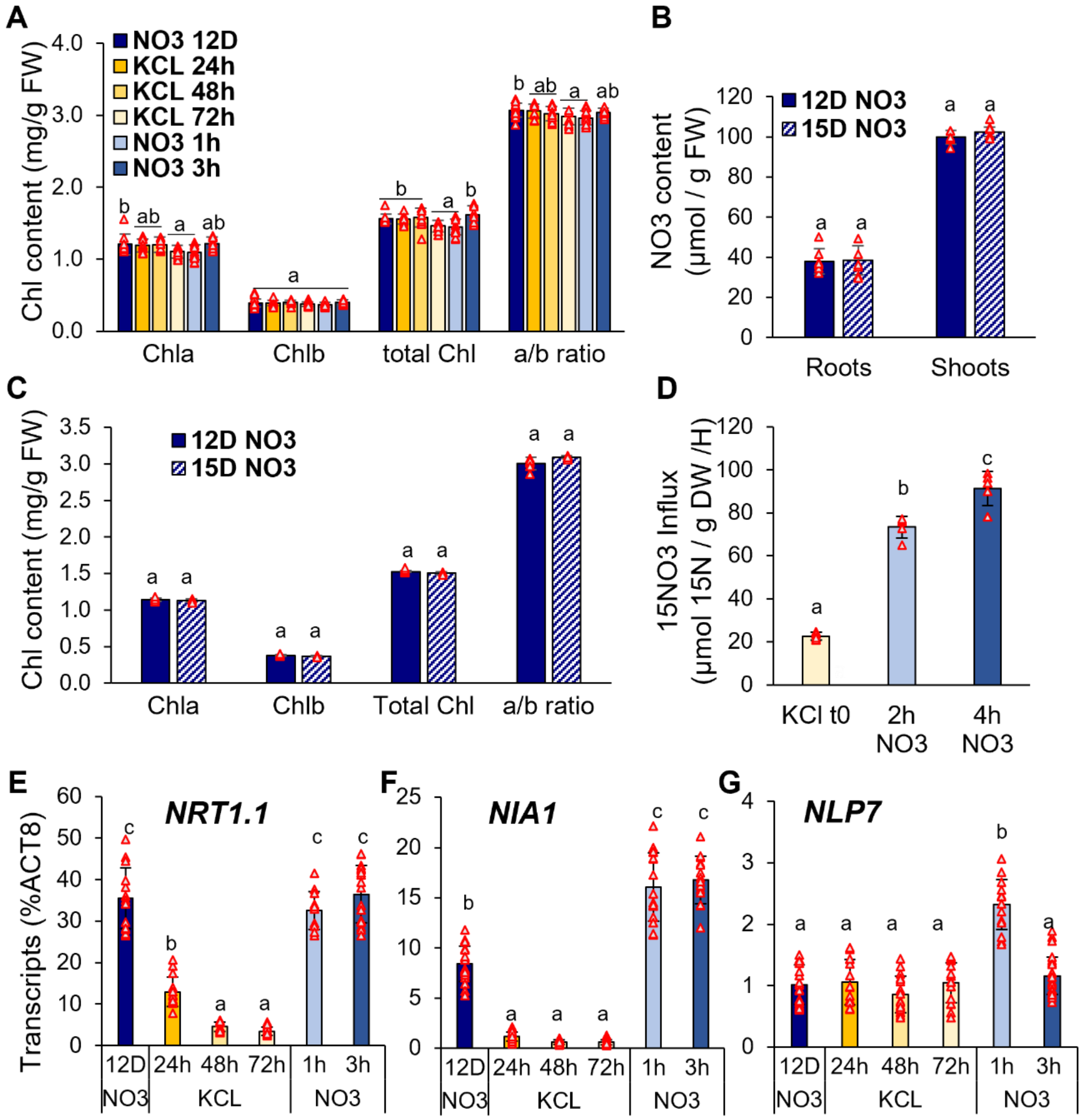

Supplemental Figure 1: Additional physiological parameters and controls [related to Figure 1].
A. Chlorophyll (chl) content profile during nitrate fluctuation kinetic. a/b ratio: the ratio between the content of chlorophyll a (Chla) and chlorophyll b (Chlb); Total Chl: sum of Chla and Chlb. **B-C.** Comparison of 12- and 15-day-old plants cultivated on constant nitrate culture for nitrate (B) and chlorophyll (C) content. **D.** Nitrate influx of 15-day-old plants cultivated for 12 days on nitrate, followed by 3 days of starvation (KCl). 5min 15N labeling was performed with plants from KCl condition (t0) or after nitrate resupply for 2h or 4h before the 5min labeling. **E-G.** Expression level of *NRT1.1* (E) *NIA1* (F) and *NLP7* (G) nitrate fluctuation. Data represent mean ± SD, N=8-12 (A), 6-7 (B), 5-6 (C), 5 (D), 8-12 (E-G). Letters indicate significant differences between time points based on ANOVA followed by post-hoc Tukey's HSD test, p<0,05.

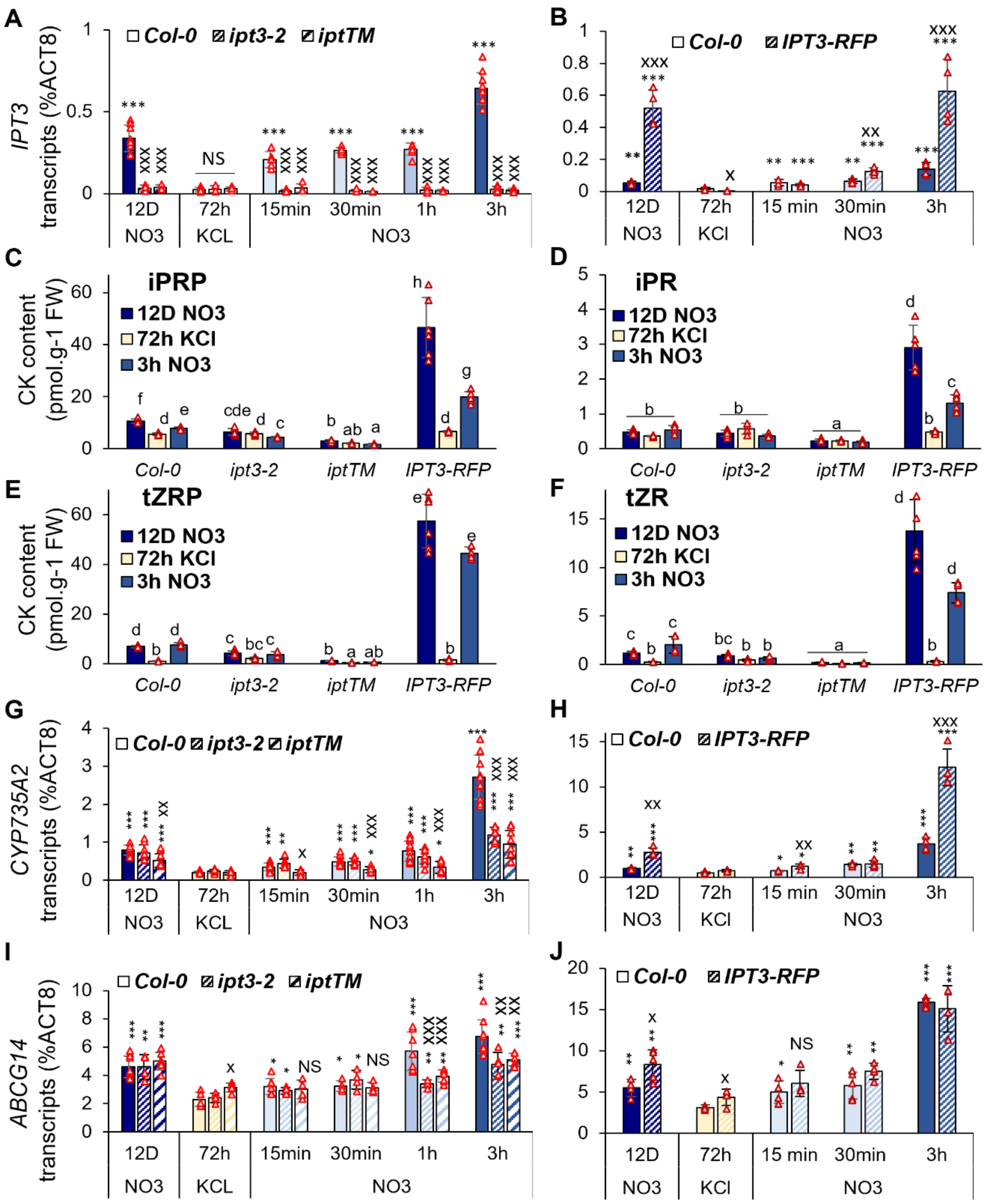

Supplemental Figure 2: Impact of IPT3 mis-regulation on iP- and tZ-precursors [related to Figure 1].

A-B. *IPT3* transcripts profile during nitrate fluctuation in *ipt3-2 and iptTM* mutant backgrounds (A) or in IPT3-RFP line (B). **C-F.** Root CK quantification of iP precursors (C-D) and tZ precursors (E-F). A one-way ANOVA test was performed, and the letters indicate significant differences based on post-hoc Tukey's HSD test, $p<0.05$. **G-J.** Transcript levels of *CYP735A2* (G-H) and *ABCG14* (I-J) during nitrate fluctuation in *ipt3-2* and *iptTM* mutant backgrounds (G and I) or in IPT3-RFP line (H and J). Data are mean ± SD, N=4-8 (A-B, G-H), 4-6 (C-F) biologically independent samples (red triangles). Asterisks denote statistically significant differences between treatments based on a two-tailed Student's *t-test* (*p<0.05, ** p<0.01, ***p<0.001, NS: non-significant). Differences between WT and mutants within each condition were performed similarly and specified by crosses (^{x} p<0,05).

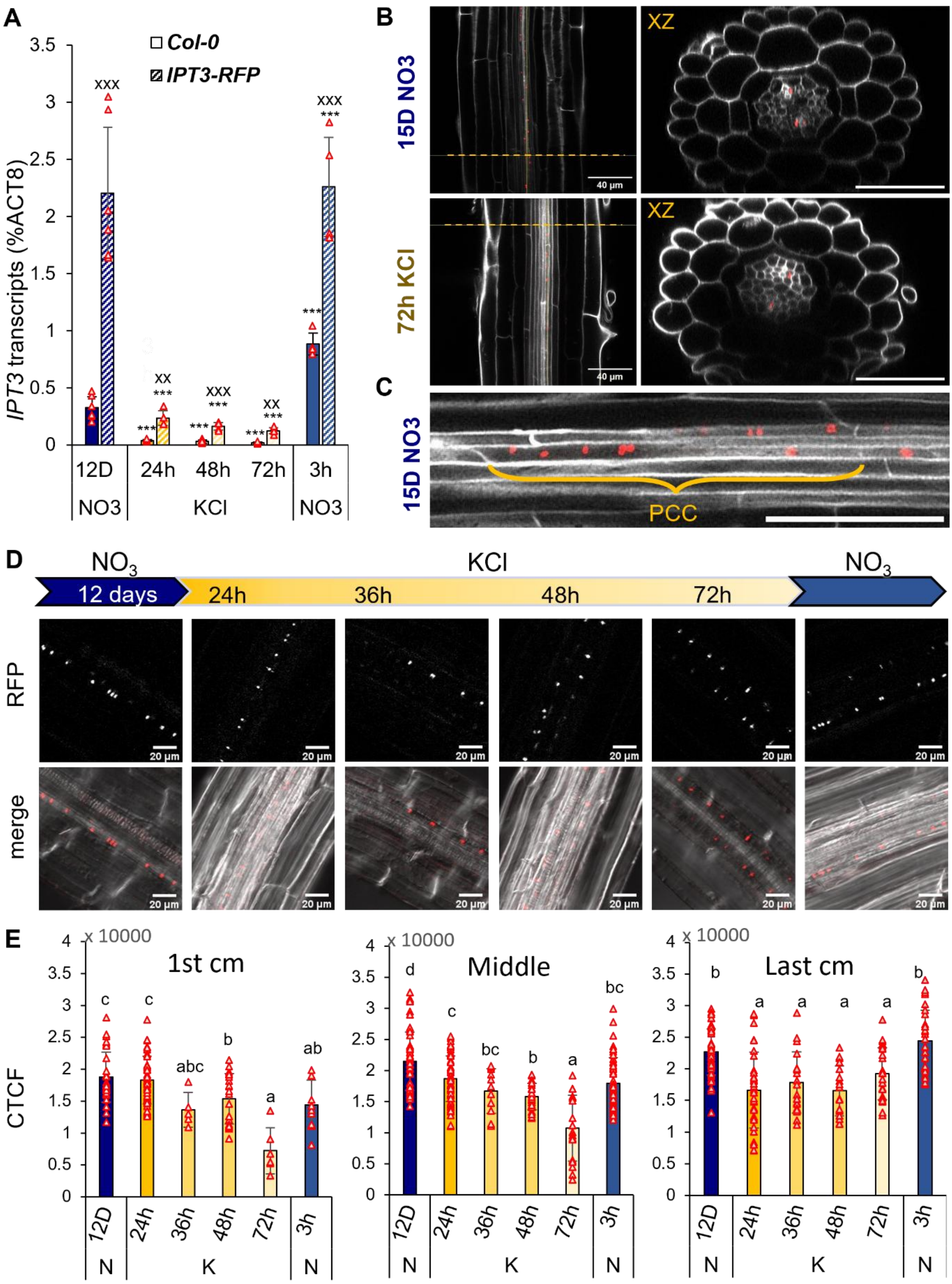

Supplemental Figure 3: Visualization of IPT3-RFP protein profile during nitrate fluctuation [related to Figure 1].

A. Level of *IPT3* expression in IPT3-RFP line compared to the WT Col-0, including during starvation. Data are means ± SD. N = 4 biologically independent samples (red triangles). Asterisks denote statistically significant differences between time points based on a two-tailed

Student's *t-test* (*p<0.05, ** p<0.01, ***p<0.001) using the NO3 condition as reference. Differences between WT and IPT3-RFP within each time point were performed similarly and specified by crosses (^{x} p<0.05). **B-C**. Location of IPT3-RFP signal inside the root on nitrate and KCl. 15-day-old plants expressing the protein IPT3 tagged by RFP (IPT3-RFP) in the *ipt357* triple mutant background were cultivated on Petri dishes. Cell walls were labeled with calcofluor white. After 3D reconstruction of the root, at 1 cm from the apex, optical cross sections (XZ) were performed at the yellow dashed line (B). The picture illustrated in C corresponds to an enlargement of a longitudinal phloem companion cell (PCC). Scale bar in both B-C = 40 um. **D-E.** Visualization (D) and quantification (E) of IPT3-RFP signal during nitrate fluctuation. Three zones of the roots were analyzed: 1 cm below the hypocotyl (1st cm), the middle of the root, and 1 cm before the root tip (last cm). All pictures represented in D were taken 1 cm before the root apex. The signal quantification corresponds to the Corrected Total Cell Fluorescence (CTCF), which was calculated as [Integrated Density – (Area of selected spot x Mean fluorescence of the background)]. Data represent mean ± SD. N = 20-50 spots over at least 12 plants. Letters indicate significant differences between time points (based on one-way ANOVA followed by post-hoc Tukey's HSD test, p<0.01).

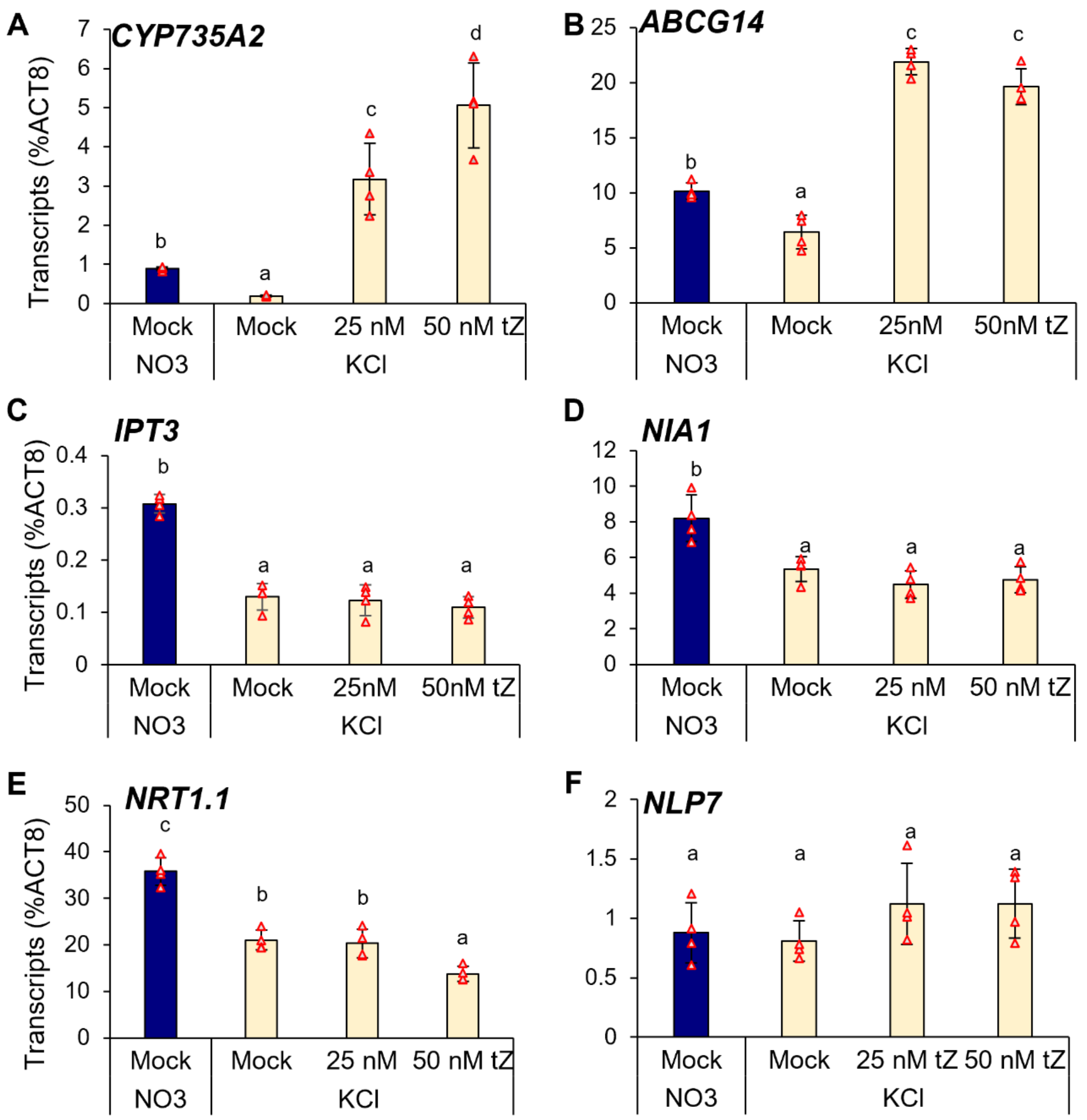

Supplemental Figure 4: Exogenous applied tZ on KCl media induced *CYP735A2* and *ABCG14* but didn't impact *IPT3* and nitrate sentinel genes [related to Figure 1].
Gene expression level of *CYP735A2* (**A**) *ABCG14* (**B**), *IPT3* (**C**) *NIA1* (**D**) *NRT1.1* (**E**) and *NLP7* (**F**) in 10 days old plants cultivated in Petri dishes first 7 days on 1mM KNO3 and then transfer 3 days on a new petri dish containing 0.01% DMSO either 1mM KNO_3 or KCl (MOCK) or KCl supplemented with 25 or 50nM of tZ. Data are mean ± SD. *N* =4 biologically independent samples (red triangles). One-way ANOVA test. Letters indicate significant differences based on post-hoc Tukey's HSD test, *p<0,05.

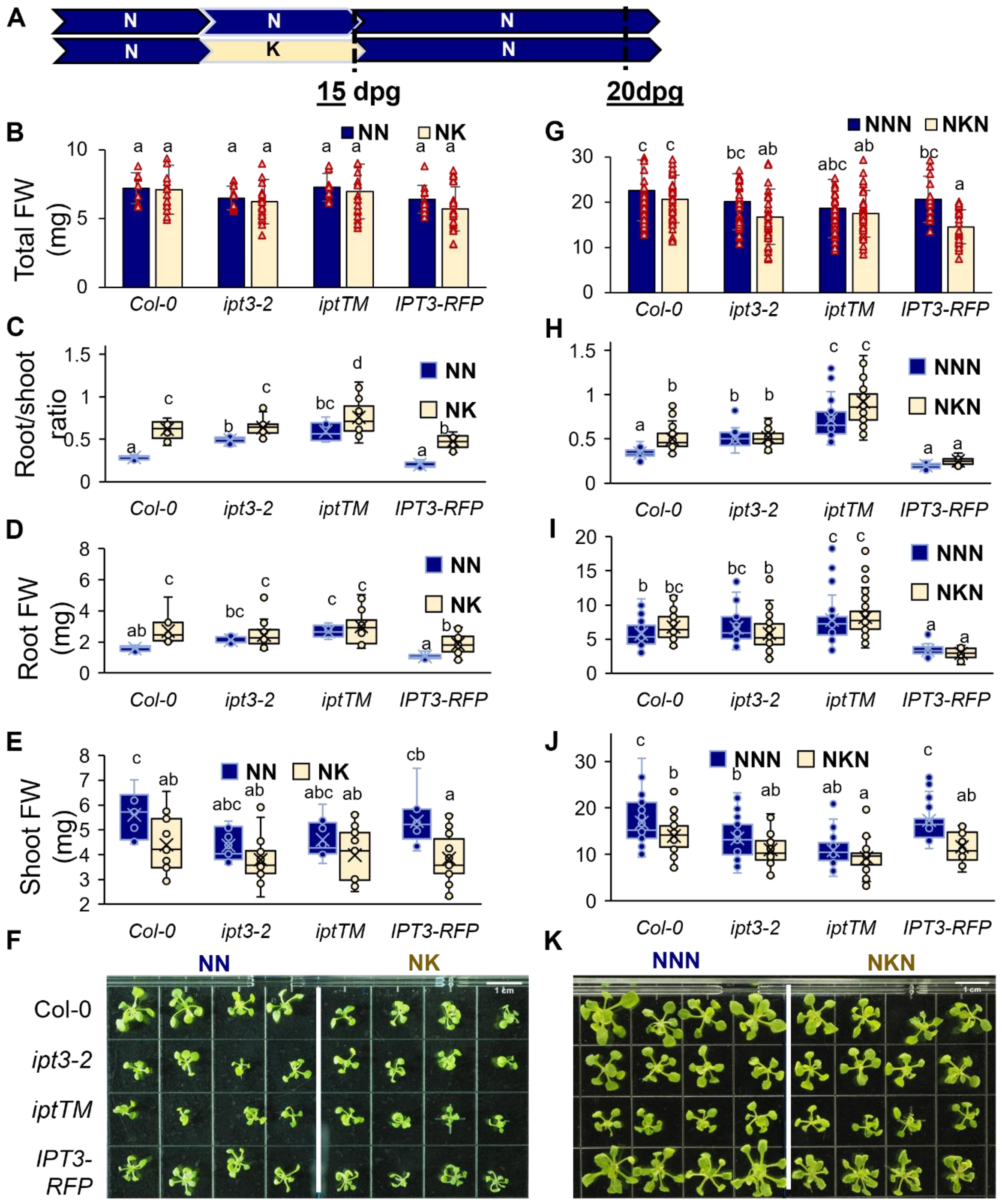

Supplemental Figure 5: growth phenotypes on Petri dishes culture [related to Figure 2]. **A.** Schematic representation of the condition names employed for nitrate variation kinetic on Petri dishes. Culture conditions are the same as in hydroponic culture where N: KNO_3 1mM, K: KCl 1mM. **B-F.** Growth phenotype at 15dpg for total FW (B), root/shoot ratio (C), root FW (D), shoot FW (E), and representative shoot pictures of 4 plants for each genotype comparing constant and fluctuating nitrate conditions (F, scale bar=1cm). **G-K.** Growth phenotype at 20dpg for total FW (G), root/shoot ratio (H), root FW (I), shoot FW (J), and representative shoot pictures of 4 plants for each genotype comparing constant and fluctuating nitrate conditions (K, scale bar=1cm). Data are mean ± SD. N=12-21 (B-E), 18-31 (G-J) individual plants grown on petri dishes. A one-way ANOVA test was performed, and the letters indicate significant differences based on post-hoc Tukey's HSD test, $p<0.05$. All boxplot

representations show inner and outlier points considered to calculate the mean, represented by the cross. The quartile was calculated by the exclusive median.

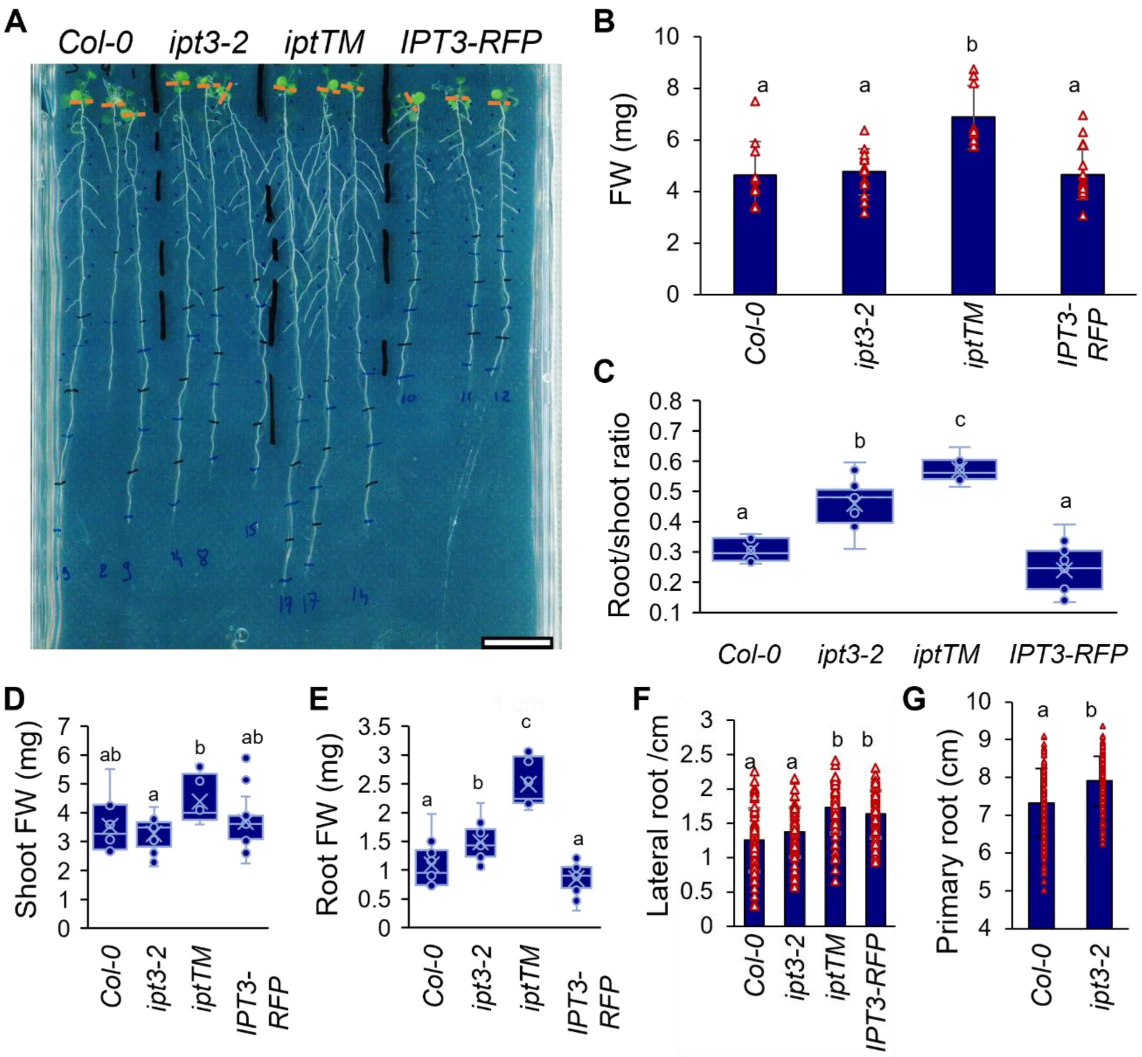

Supplemental Figure 6: Details of growth phenotypes at 12dpg in Petri dish culture on constant nitrate [related to Figure 2].

A. Picture of a representative agar plate showing all genotypes at 12dpg (scale bar=1cm). Orange dashed lines highlight the starting of the primary root and blue or black lines along the primary root show the daily length measurement since 7dpg corresponding to plants' transfer on a fresh nitrate plate. Blue dots and the number below plants correspond to the number of lateral roots counted. Picture brightness and contrast were slightly increased for better root visualization. **B.** Seedling FW after 12 days post germination (dpg). **C.** Root/shoot ratio **D.** Shoot FW. **E.** Root FW**. F.** Lateral root density. **G.** Primary root length phenotype of *ipt3-2*. Data are mean ± SD. N=12-19 (B-E), 57-62 (F), 112-121 (G) individual plants grown on Petri dishes. A one-way ANOVA test was performed, and the letters indicate significant differences based on post-hoc Tukey's HSD test, p<0.05. All boxplot representations show inner and outlier points considered to calculate the mean, which is represented by the cross, quartile was calculated by the exclusive median.

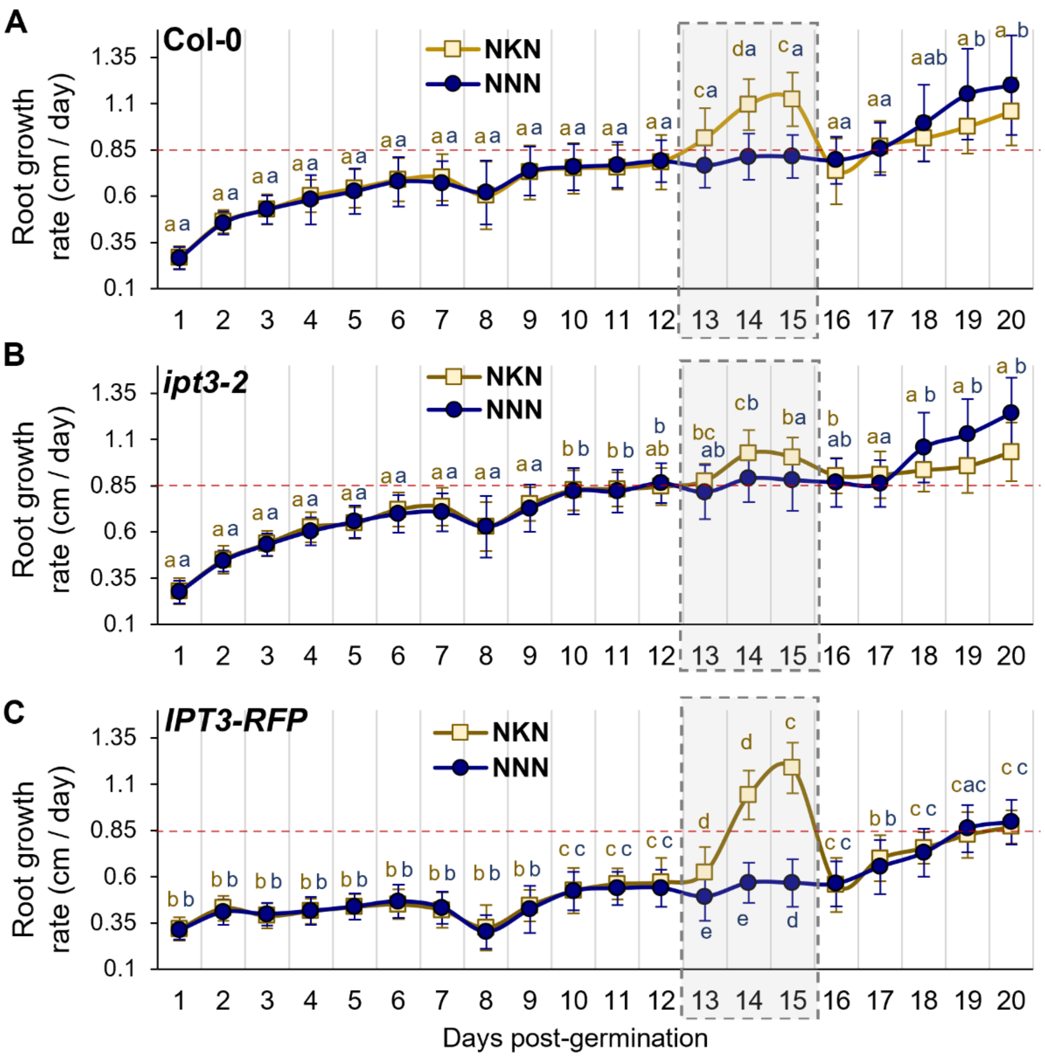

Supplemental Figure 7: Complete kinetic of the primary root growth rate [related to Figure 2G-H].

Rate of primary root growth corresponding to the increase in length every 24h, in Col-0 (**A**), *ipt3-2* (**B**), and *IPT3-RFP* (**C**) Data are mean ± SD. N = 35-70 individual plants. A one-way ANOVA was performed to compare genotype and treatment in each time point separately where blue letters correspond to the NNN condition and orange letters correspond to the NKN condition. A red dashed line was added as visual support facilitating the comparison between the genotypes.

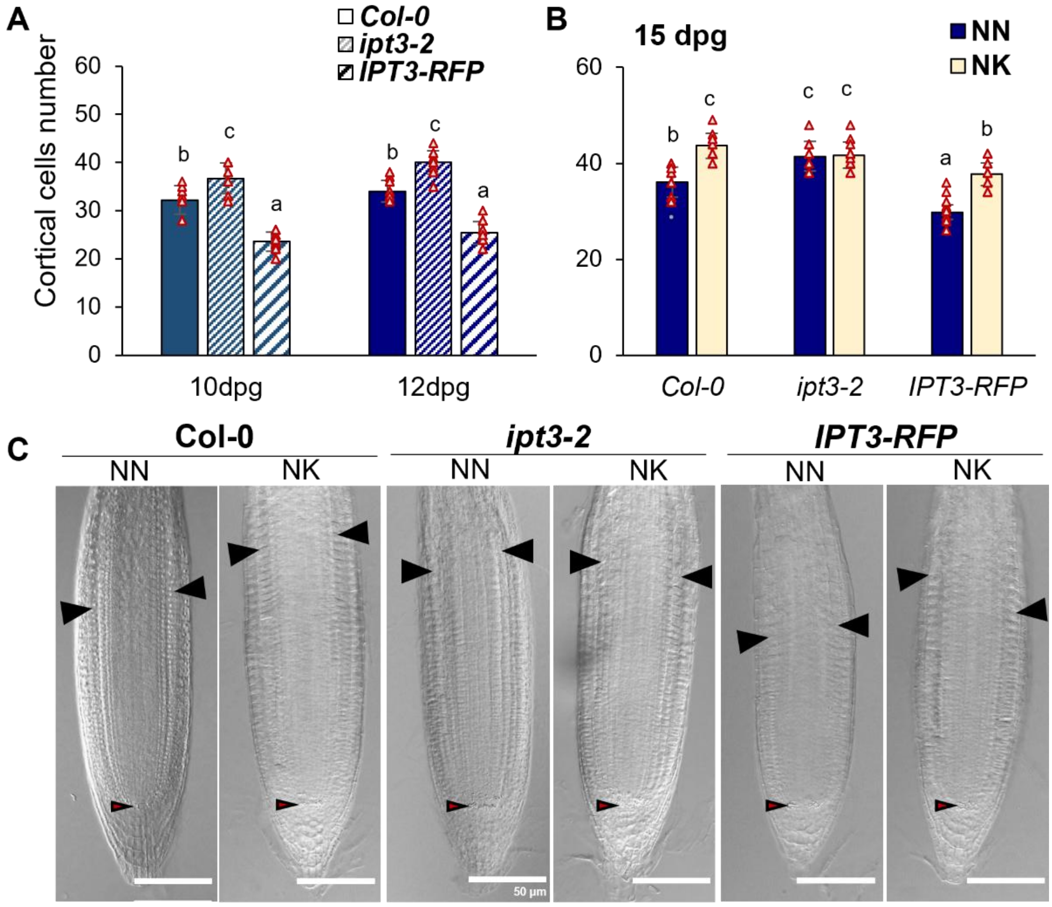

Supplemental Figure 8: *IPT3* is important for the nitrate-dependent limitation of meristem size [related to Figure 2G-H].

A-B. Number of cortical cells in the root meristematic zone during the culture process at 10- and 12-days post germination (dpg) on nitrate (A) and at 15dpg (B) after 3 days transfer on nitrate (NN) or KCl (NK). Data are mean ± SD. N=10-17 (A), 10-15 (B), individual plants (red triangles) grown on petri dishes. A one-way ANOVA test was performed, and the letters indicate significant differences based on post-hoc Tukey's HSD test, $p<0.05$. **C.** Representative pictures of meristem size in all genotypes at 15dpg. Scale bar=50um. The red arrow points to the quiescent center and the black arrows to the end of the meristematic zone, characterized by an increase in cortical cell size (doubling of volume).

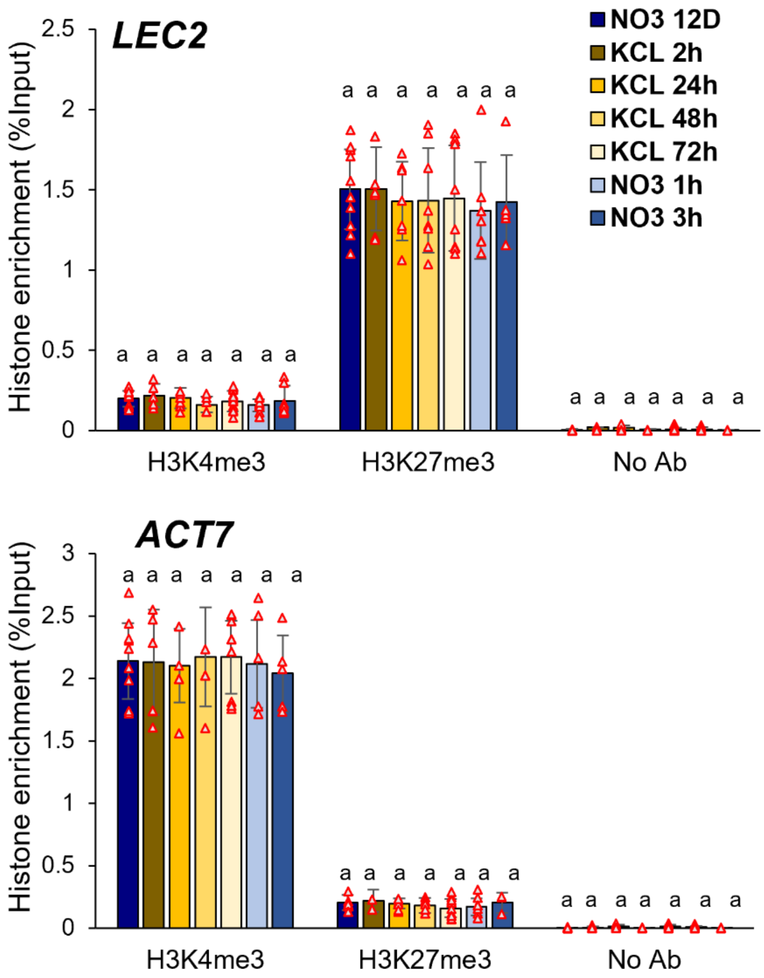

Supplemental Figure 9: Chromatin profile of two control genes during nitrate fluctuation [related to Figure 3].

Histone enrichment of 2 control genes at a similar position to *IPT3* GB1. *LEC2* is a positive control for H3K27me3 and a negative control for H3K4me3, while *ACT7* is a negative control for H3K27me3 and a positive control for H3K4me3. Data are mean ± SD. *N* =5-10 biologically independent samples (red triangles). A one-way ANOVA test was performed, and the letters indicate significant differences based on post-hoc Tukey's HSD test, $p<0.05$.

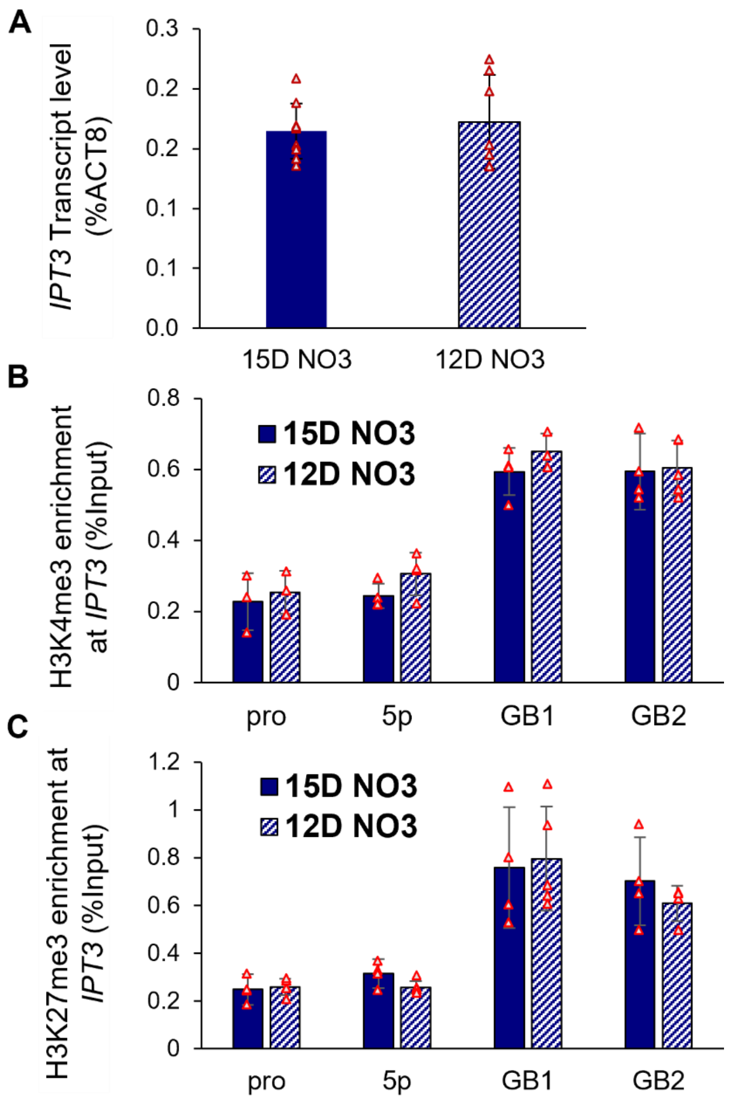

Supplemental Figure 10: On constant nitrate culture, *IPT3* transcript and chromatin profiles are stable. [related to Figure 3].

A. Comparison of *IPT3* expression profiles between plants cultivated on constant nitrate culture for 12 (striped colors) and 15 days (full colors). No significant differences were obtained (Student's *t-test*). Data represent mean ± SD. N = 6 biologically independent samples (red triangles). **B-C.** Comparison of 12 d or 15d constant nitrate culture for H3K4me3 (B) and H3K27me3 (C) enrichment at *IPT3* loci. Data represent mean ± SD. N = 3-5 biologically independent samples (red triangles). No significant differences were obtained (Student's *t-test* and ANOVA).

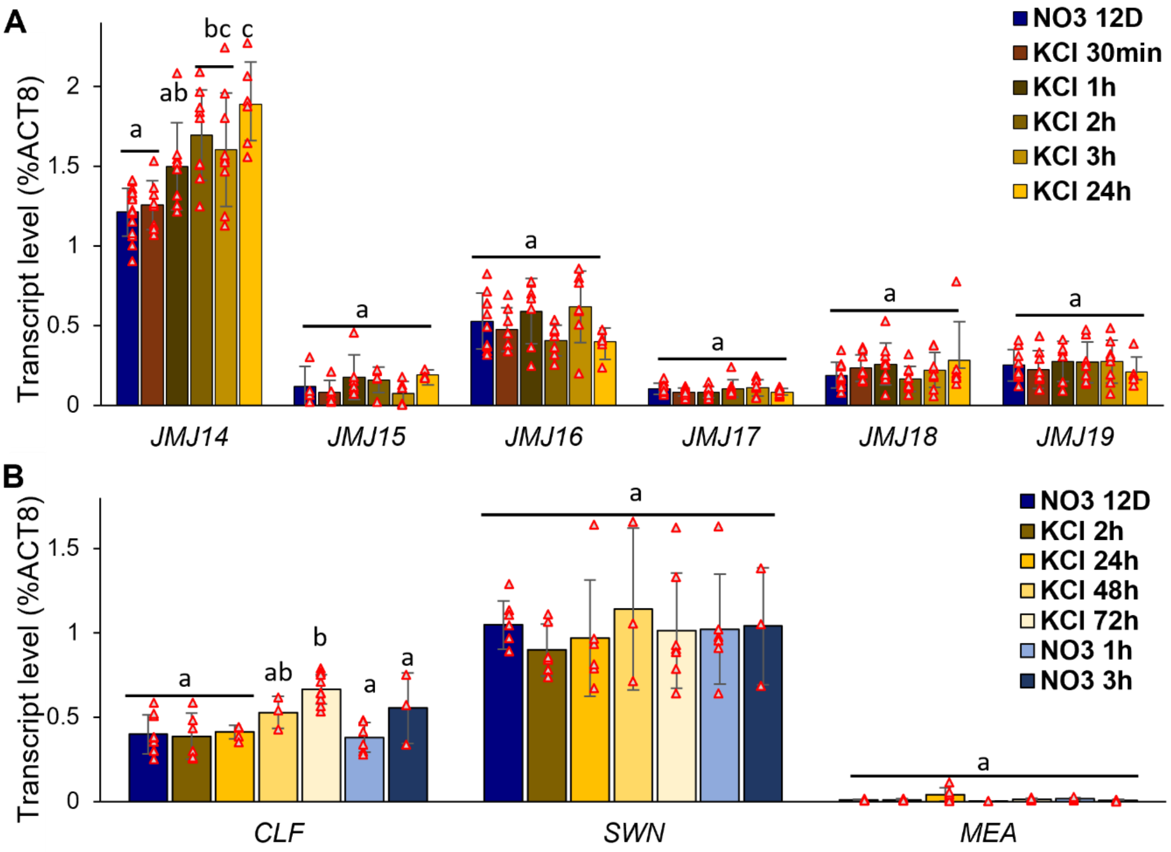

Supplemental Figure 11: Expression profile of H3K4me3 demethylases and H3K27 methyltransferase during N starvation [related to Figure 3 and Figure 4].

A. Expression profile of KDM5 subfamily *JUMANJI* (*JMJ*) genes involved in H3K4me3 demethylation. **B.** Expression profile of Polycomb Repressive Complex 2 tri-methyltransferase *CURLY LEAVES* (*CLF*), *SWINGER* (*SWN*), and *MEDEA* (*MEA*). 12-day-old plants were cultivated in hydroponic media containing nitrate, followed by starvation (KCl) for a few minutes to 24 hours. Data represent mean ± SD. N = 5-15 (A), 3-6 (B) biologically independent samples (red triangles). A one-way ANOVA test was performed, and the different letters indicate significant differences based on post-hoc Tukey's HSD test, $p<0.05$.

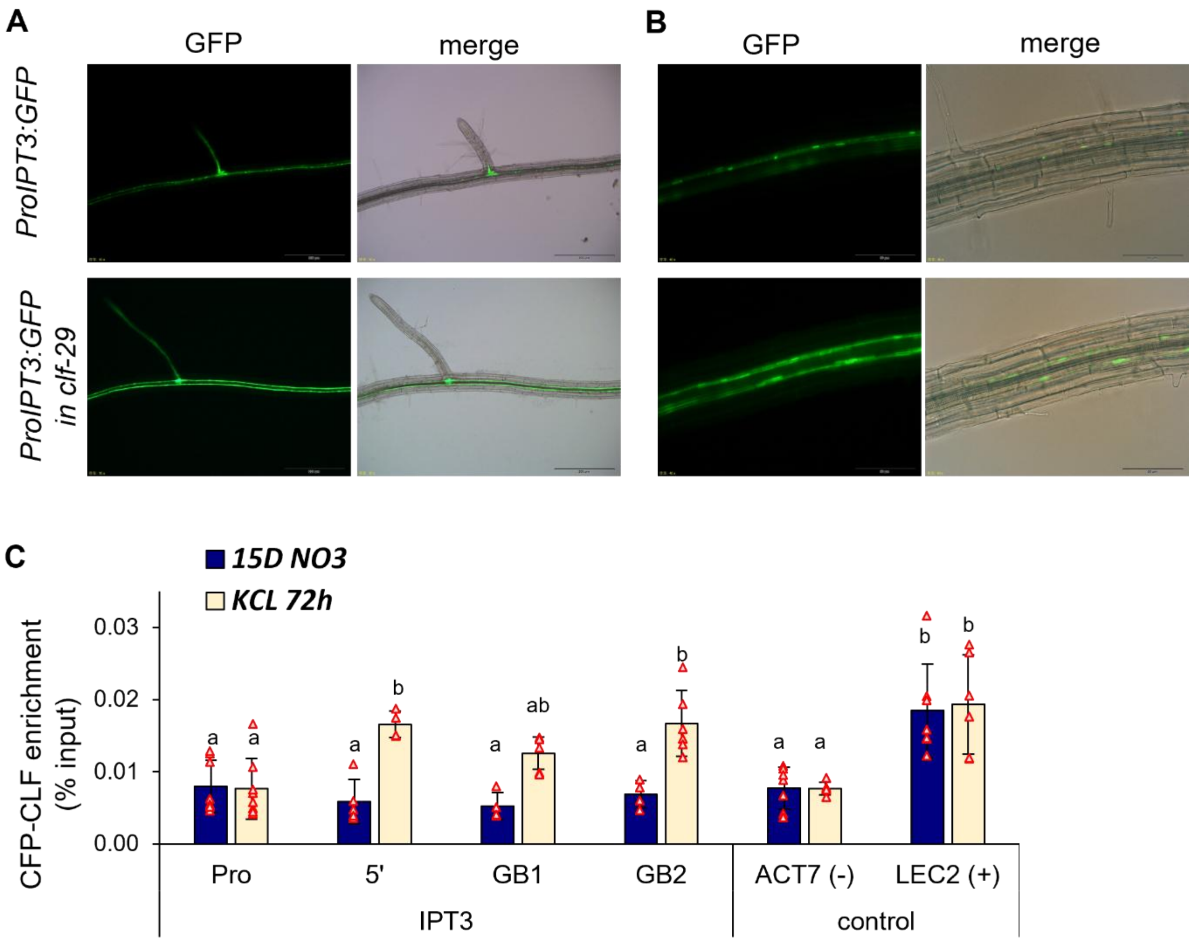

Supplemental Figure 12: CLF is involved in *IPT3* repression in phloem companion cells on KCl [related to Figure 4].

A-B. Representative pictures taken at a large scale (A, scale bar = 200um) and closer view (B, scale bar = 50um) of 15-day-old plants cultivated on nitrate and expressing *pIPT3::GFP* in WT and *clf-29* mutant. A stronger but not ectopic expression of *IPT3* is observed in the *clf-29* mutant. All pictures were taken in a similar region at the middle of the primary root. **C.** CLF enrichment at *IPT3* locus related to the input. ChIP experiments were performed with chromatin from roots of 15-day-old *clf-29* plants carrying a *ProCLF:CFP:CLF* transgene, grown under constant nitrate (1 mM NO3, 15 days) or 12 days on nitrate followed by 3 days KCl (KCl 72h). ChIP was performed using GFP-trap antibodies, quantified by qPCR, and normalized by the input. LEC2 (positive control, +) and ACT7 (negative control, -). Data are mean ± SD. N = 5-8 biologically independent samples (red triangles).). A one-way ANOVA test was performed, and the letters indicate significant differences based on post-hoc Tukey's HSD test, $p<0.05$.

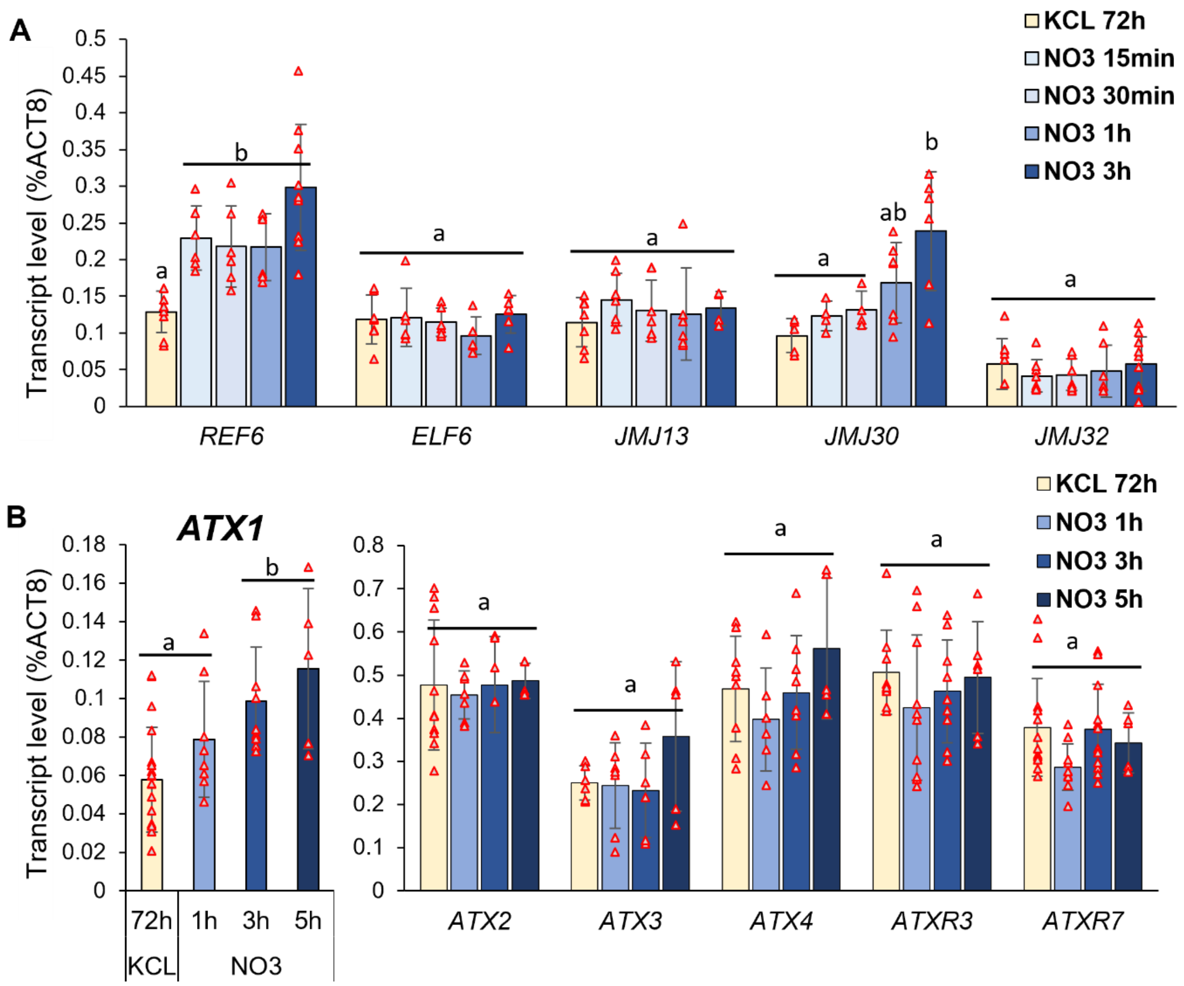

Supplemental Figure 13: Expression profile of H3K27me3 demethylases and H3K4 methyltransferase during N starvation [related to Figure 3 and Figure 5].

A. Expression profile of JMJ protein involved in H3K27me3 demethylation. **B.** Expression profile of ARABIDOPSIS TRITORAX-like (ATX) and ATX-Related (ATXR) involved in H3K4 di- and tri-methylation. 15-day-old plants were cultivated in hydroponic media containing nitrate for 12 days followed by 3 days of starvation (KCl 72h) before resupplying nitrate for few minutes to 3 hours. Data are mean ± SD. N = 5-15 (A), 3-6 (B) biologically independent samples (red triangles). A one-way ANOVA test was performed, and the different letters indicate significant differences based on post-hoc Tukey's HSD test, $p<0.05$.

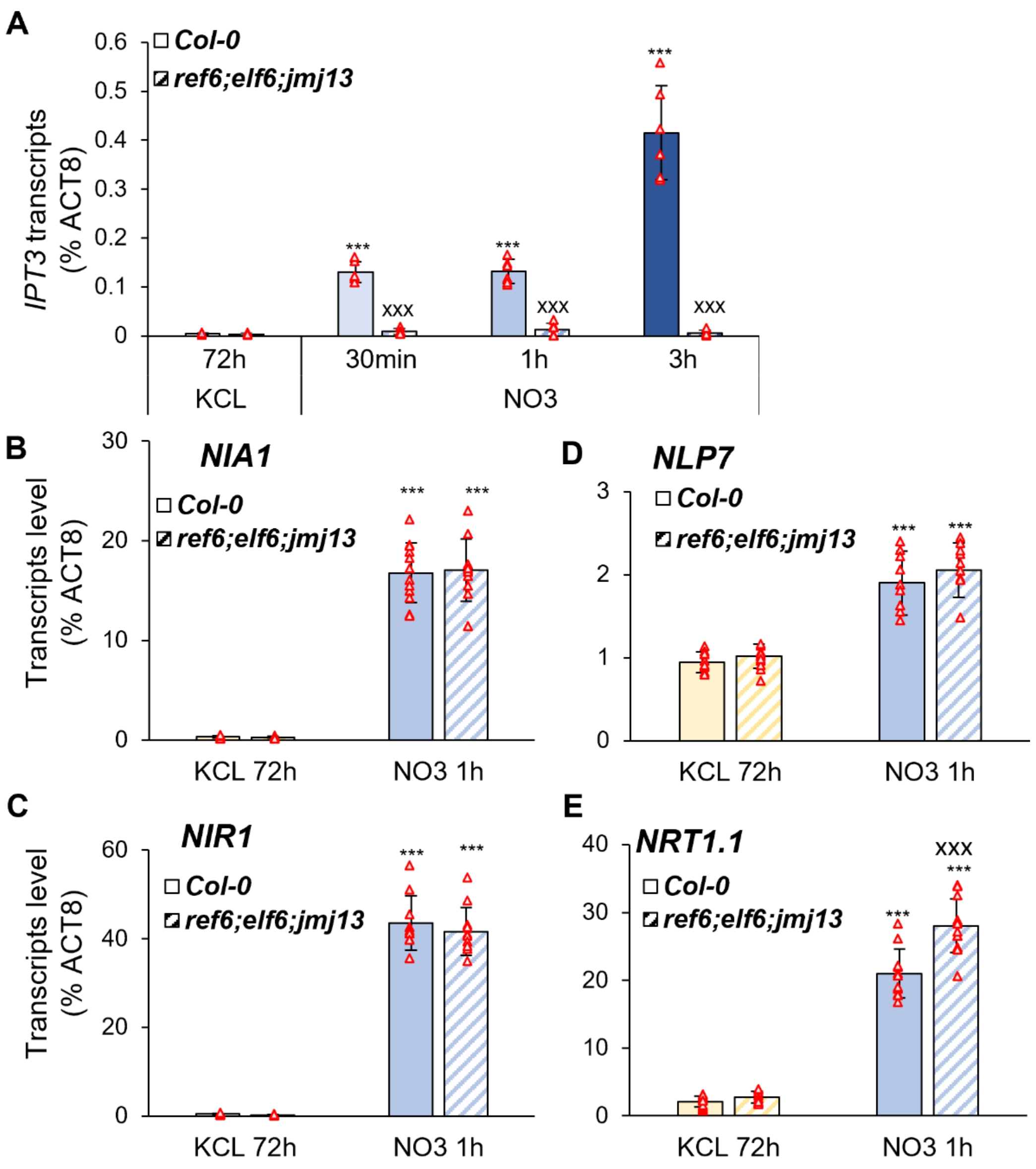

Supplemental Figure 14: H3K27me3 demethylases are not involved in nitrate sentinel genes induction by nitrate [related to Figure 5].
Impact of *ref6 elf6-3 jmj13* triple-mutant on gene induction. Two independent experiments showed a lack of *IPT3* induction (**A**) while nitrate sentinel genes (**B-E**) are induced properly: *NIA1* (B), *NIR1* (C) *NLP7* (D), and *NRT1.1* (E). 15-day-old plants were cultivated in hydroponic media containing nitrate for 12 days, followed by 3 days of starvation (KCl 72h) before resupplying nitrate for 1h. The color code is based on time points as illustrated in Figure 1A, with full colors for Col-0 and striped colors for mutants. Data are mean ± SD. N = 6 (A), 9-13 (B-E) biologically independent samples. Asterisks denote statistically significant differences between KCl and other time points based on a two-tailed Student's *t-test* (* p<0,05, ** p<0,01, *** p<0,001). Differences between WT and mutants within each time point were performed similarly and specified by crosses (^{x} p<0,05).

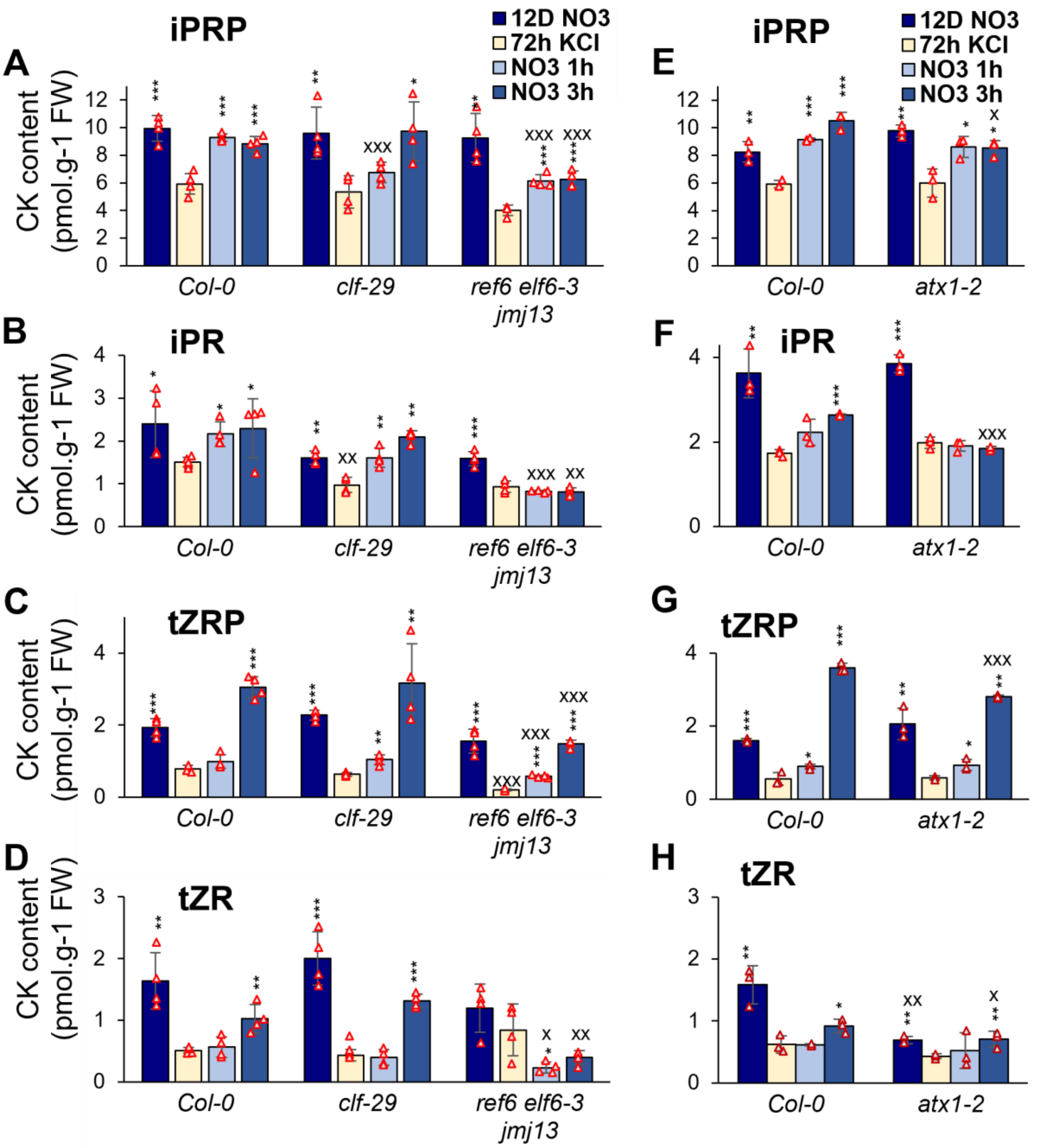

Supplemental Figure 15: Impact of chromatin mutants on iP- and tZ-precursors [related to Figure 6].

Root CK profiles during nitrate fluctuation in H3K27 related mutants *clf-29* and triple mutant *ref6 elf6-3 jmj13* (**A-D**) or in *atx1-2* mutant (**E-H**). Data are mean ± SD. N = 4-5 (A-D), 3 (E-H), biologically independent samples (red triangles). Asterisks denote statistically significant differences between KCl and other time points based on a two-tailed Student's *t-test* (* p<0.05, ** p<0.01, *** p<0.001). Differences between WT and mutants within each time point were performed similarly and specified by crosses (^{x} p<0.05).

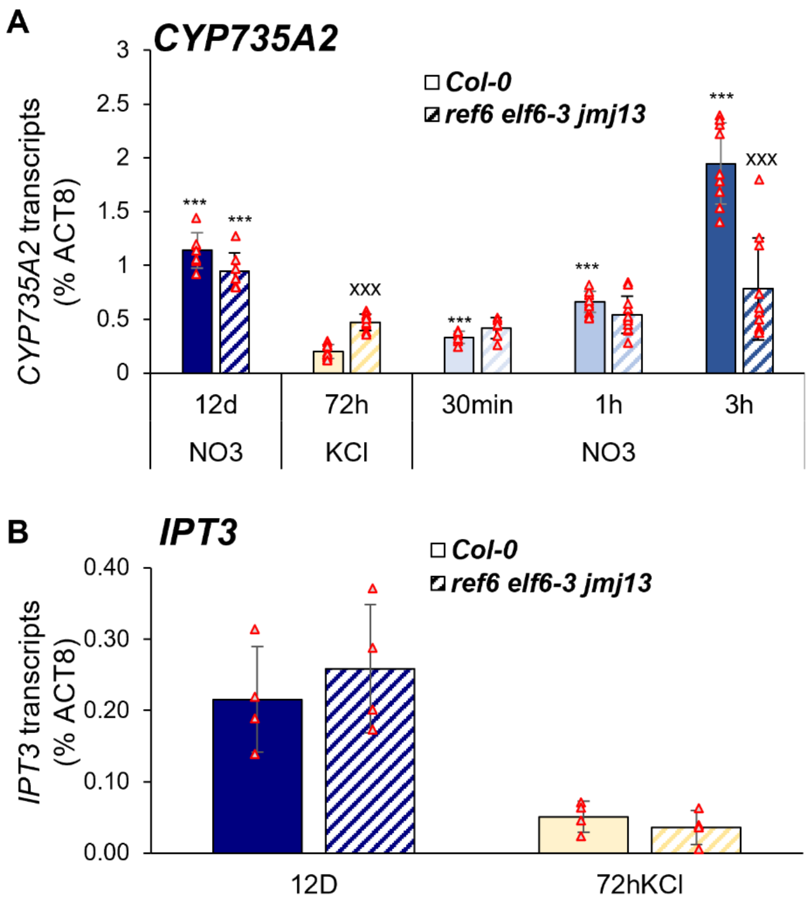

Supplemental Figure 16: Additional expression in *ref6 elf6 jmj13* triple-mutant.

Impact of *ref6 elf6-3 jmj13* triple-mutant on *CYP735A2 (**A**)* and *IPT3* (**B**) transcripts during nitrate fluctuation. The color code is based on time points as illustrated in Figure 1A, with full colors for Col-0 and striped colors for mutants. Data are mean ± SD. N = 7-11 (A), 4 (B) biologically independent samples (red triangles). Asterisks denote statistically significant differences between KCl and other time points based on a two-tailed Student's *t-test* (* p<0.05, ** p<0.01, *** p<0.001). Differences between WT and mutants within each time point were performed similarly and specified by crosses (^{x} p<0.05).

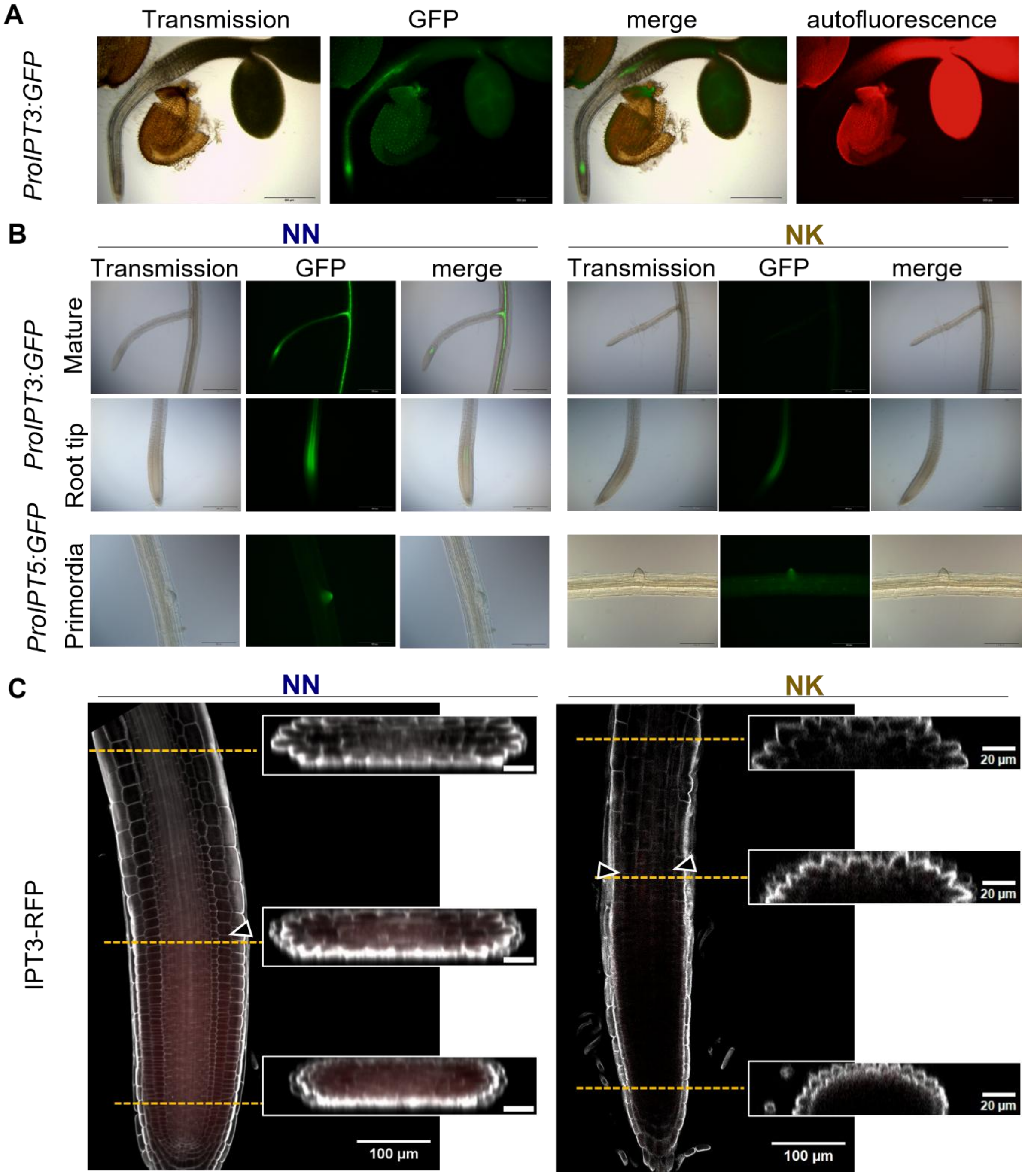

Supplemental Figure 17: Location of *IPT3* and *IPT5* expression is different [supporting discussion].

A-B. Visualization of GFP expression pattern under the control of *IPT3* or *IPT5* promoter. 24h post germination transgenic seedling (1dpg) expressing *proIPT3::GFP* (A) and 15 days post germination transgenic roots (15dpg) expressing *proIPT3::GFP* or *proIPT5::GFP* (B) grown under constant nitrate (NN) or fluctuating nitrate (NK) culture as illustrated in Figure 1A. **C.** Visualization RFP signal in IPT3-RFP line at the root tip depending on the condition explained in B. XZ optical section were performed at similar location of the root : elongation zone, just before cortical cells ‘elongation illustrated by the arrow, and in the meristematic zone. For all panels (A-C), at least 5 plants were observed. Scale bar = 200 µm (for *ProIPT3:GFP* in A-B), 100 µm (for *ProIPT5:GFP* in B and IPT3-RFP in C), and 20 µm XZ optical sections in C.

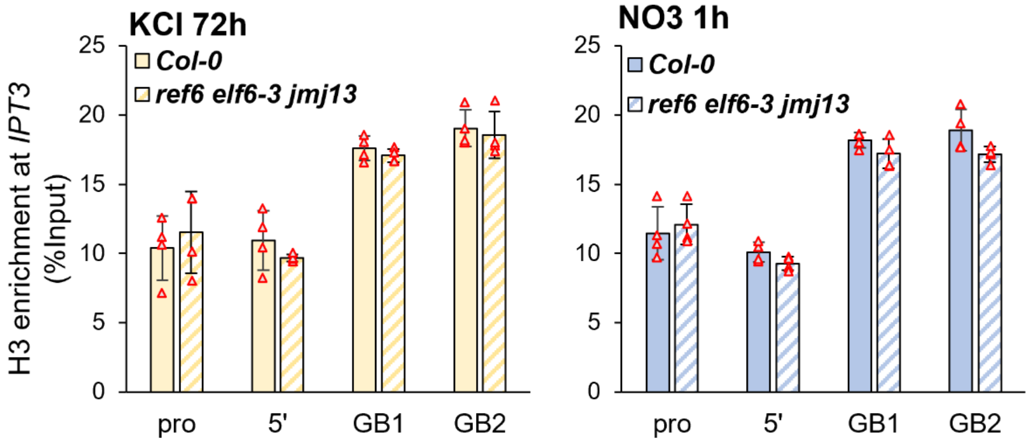

Supplemental Figure 18: H3 level at *IPT3* is not impacted in the mutant for H3K27me3 demethylase [supporting discussion]*.*

H3 enrichment at the 4 regions of the *IPT3* locus was analyzed by ChIP-qPCR on 15-day-old plants after 3 days of starvation (left) and after 1h of nitrate (right) in Col-0 (full color) *and ref6 elf6-3 jmj13* mutant (striped colors). Data are mean ± SD. N = 4 biologically independent samples (red triangles). No significant differences were obtained for either treatment or mutant effect (two-tailed Student's *t-test*). Pro: promoter, 5': inside the 5' UTR, GB: Gene body, as illustrated in Figure 3A.

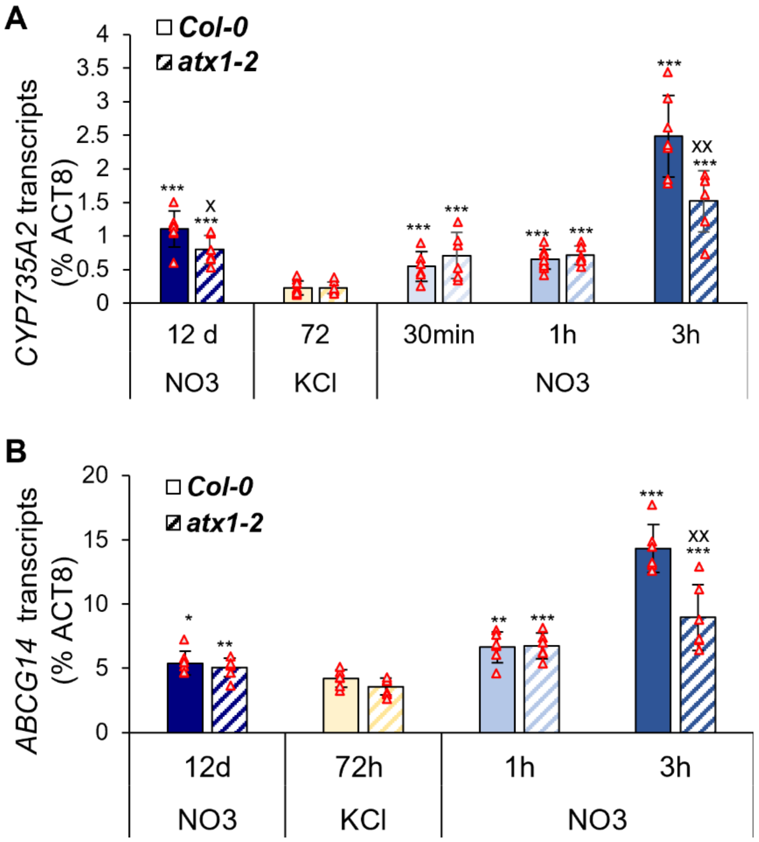

Supplemental Figure 19: *CYP735A2* and *ABCG14* expression in *atx1-2* mutant is reduced after 3h of resupply [related to Figure 6].

Impact of *atx1-2* mutant on transcript levels of *CYP735A2* (**A**) and *ABCG14* (**B**). The color code is based on time points as illustrated in Figure 1A, with full colors for Col-0 and striped colors for mutants. Data are mean ± SD. N = 6-7(A), 7-9 (B) biologically independent samples (red triangles). Asterisks denote statistically significant differences between KCl and other time points based on a two-tailed Student's *t-test* (* p<0,05, ** p<0,01, *** p<0,001). Differences between WT and mutants within each time point were performed similarly and specified by crosses (^{xx} p<0,01).

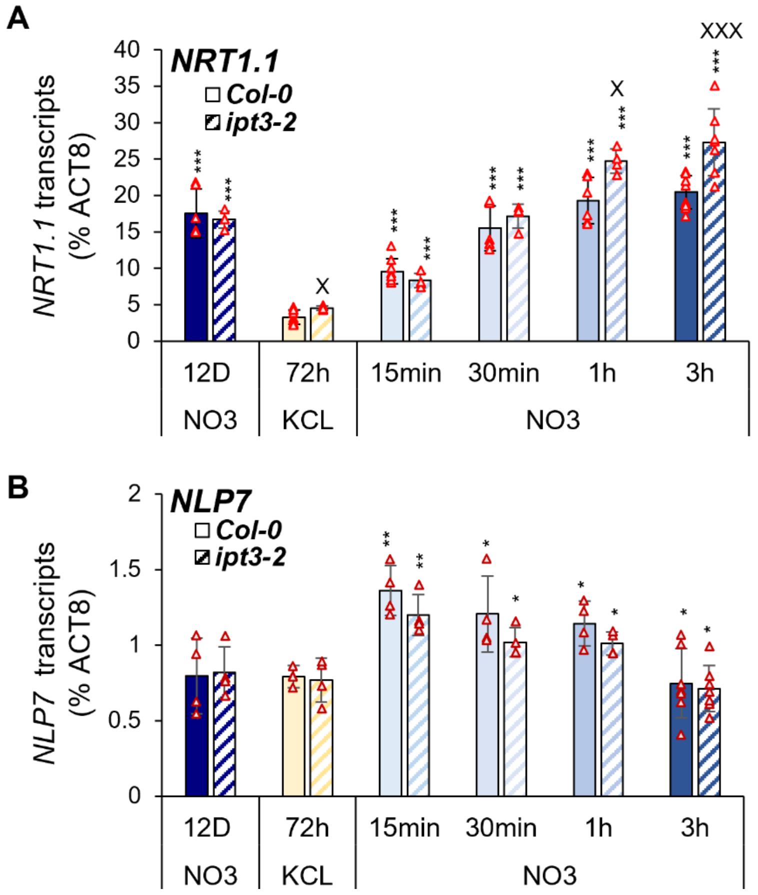

Supplemental Figure 20: Nitrate sensors are induced by nitrate in *ipt3-2.*
Impact of *ipt3-2* mutant on transcript levels of *NRT1.1* (**A**) and *NLP7* (**B**). The color code is based on time points as illustrated in Figure 1A, with full colors for Col-0 and striped colors for mutants. Data are mean ± SD. N = 4-8 biologically independent samples (red triangles). Asterisks denote statistically significant differences between KCl and other time points based on a two-tailed Student's *t-test* (* p<0,05, ** p<0,01, *** p<0,001). Differences between WT and mutants within each time point were performed similarly and specified by crosses (^{x} p<0,05).

Gene name	AGI	Forward/ Reverse	Sequence	Application
RFP	-	F	tctagtggcgatggtgtctaagggcgaag	Cloning
		R	caagaaagctgggtctagattcaattaagtttgtgcccc	
IPT3	AT3G63110	proIPT3-F	ctttaaaggaaccaattcagttgccaagagttcaagcgaac	Cloning
		IPT3CDR-R	tagacaccatcgccactagacaccgcga	
		pro_F	GACCGATCATGAGGAGGAAG	ChIP-qPCR
		pro_R	AACTCGGCAATTACCTTGGA	
		5'UTR_F	ATTGCACGCGGATCAAATA	
		5'UTR_R	AATGGCCACTGTACGAATGA	
		GB1_F	AGTCACGACTCTCCGTGGAT	
		GB1_R	CTCTCCTCGCTCGTGATCTT	
		GB2_F	CATGGCGAATCTCTCCATTGA	ChIP-qPCR & RT-qPCR
		GB2_R	AGTTGGAACCTCCAACGATGA	
ACT7	AT5G09810	F	GCATGCGTTGTGGTTTTATG	ChIP-qPCR
		R	AGCGAGAGATCGACAGAAGC	
LEC2	AT1G28300	F	CGCTCGCACTTCACAACAGTCC	ChIP-qPCR
		R	TCATCACCGCCGCCATCTGC	

Supplemental Table 1: Primers used for cloning and ChIP-qPCR and their applications.

Gene name	AGI	Forward sequence	Reverse sequence
ACT8	AT1G49240	GGATCTCTAAGGCAGAGTATGA	TCTCCAAACGCTGTAACCGGA
NRT1.1	AT1G12110	GCACATTGGCATTAGGCTTT	CTCAATCCCCACCTCAGCTA
NLP7	AT4G24020	TTCTCCGACGGTGGAGGAAATG	CAGCTGCTGATGGAGAAGAGTAAG
NIA1	AT1G77760	GGCTACGCTTATTCTGGAGGAGGT	TGGTGGTCAAGCTCACAAACACTC
NIR1	AT2G15620	AACCGTTTCTCCCCTGAACC	CTTGTCCGCAGAACTGGCTA
ABCG14	AT1G31770	TCGGTGCTCTGCTTATGAAC	ACGATGAAGGGAGGAATTTG
CYP735A2	AT1G67110	ATGGTGTCCCTTCCGTTGAACA	GAGGGTAAAGTCTTAATGACTCGT
IPT5	AT5G19040	AGGATTTTCAGCGTGAAGCAA	CTATGATCGGGACACGGTCTCT
IPT7	AT3G23630	AACCTAACGGCCACCCAGTA	TGTTGTTCGCTGAGAGTTTCGA
CLF	AT2G23380	TTGTTTGCTAAACGGGACTTGCTG	TTCTTGCAGCTCTTTGGGCAACC
MEA	AT1G02580	GCTGCTAATCGTGAATGCGATCC	AGAGTGCCATCTCCACAGCTAAG
SWN	AT4G02020	ACCCAATTGCTACGCTAAGGTG	GCTAGCTTCTATTCGTTCGTTTGC
JMJ14	AT4G20400	TCTTGGTCCATTGTTCAGGGTCTC	TTGTGTCACCATTTGCCAGCAC
JMJ15	AT2G34880	AGAGGCCAGACATTGGTGAAGC	AGCAAGCGTATCTTCAAACTCCTC
JMJ16	AT1G08620	TGTGCCGTGGCTGTATATTGGG	ACAAGTGGTGATCTTCAACATGCC
JMJ17	AT1G63490	TCTAGCTCACCTACCTCTGAGTTG	AGTGCTGCTGAATATGCTGTAACG
JMJ18	AT1G30810	AGTCCACGTCATCGGAAGGTTG	AATTGGCCTCTGTGCTTCATCAG
JMJ19	AT2G38950	AGTCCTTGAGGAAGCTCCTGTG	TGAGAGTGTGTCCCTGAATTCCTC
ATX1	AT2G31650	ATGCCGAGACAAAGACTCATCCG	TGGAGTAGGCCCTTTGAACTGCTC
ATX2	AT1G05830	GAGTGGCATATCATCCGCTATGTG	GCCTATCCTCATCTGCAAGCTC
ATX3	AT3G61740	ACGTATGGGTACATGCTGAATGCG	TGTTGTGCTCCAGTTCCTTAAAGC
ATX4	AT4G27910	GTGGACTATGTTGCCAGGTTCC	TCTCCGTGAATCCCTGATCTGC
ATXR3	AT4G15180	TGCTTGGTGGGTTGCCAGATTG	CTCGAAATTGATGAACCGGACCAG
ATXR7	AT5G42400	AGGCTTGACGATGGCTATGTGC	TGATAAACCTCGCTATGCCACCTC
REF6	AT3G48430	CCGGAATACCGTGTTGCAGGTTAG	TCCCGGAAAGGTGACGACAAAC
ELF6	AT5G04240	TCTACCCATGCTGTCCCATCAG	AGCAATGATCGTGGCACTCTTG
JMJ13	AT5G46910	TGAAACATCCATTCCGGGAGTCAC	CCTCAACATGCCAGGCAAACATAC
JMJ30	AT3G20810	GCTGGGACAGTTACTCCGTTACAC	ACTTCTTGCCAACAACCTGAGC
JMJ32	AT3G45880	TCCGACTGATGTTCACCGTCTC	AGCTTGAACGCATCAGTATCTCG

Supplemental Table 2: RT-qPCR primers used in this study. Primers for *IPT3* are listed in Supplemental Table 1.

Supplemental material and methods

Chlorophyll content

About 20 mg of entire shoots were incubated in 2mL DMF for about 24h at 4°C in the dark (shaking occasionally until leaves appear white). As much as possible, samples were kept in the dark and cold to protect chlorophyll from light damage. The absorbance of each sample was measured at 647 and immediately after at 665nm using a quartz cuvette. The calculation was performed according to the Inskeep and Bloom, 1985[S1] equation, but included sample volume and weight to have results in mg/g FW instead of mg/ml.

Chl a = [12.70A665 - 2.79A647]*x V/(1000 x FW)*

Chl b = [20.70 A647 - 4.62A665]*x V/(1000 x FW)*

total Chl = [17.90A647 + 8.08A665]x V*/(1000 x FW)*

where V=volum extract FW=fresh weight

Influx

15-day-old plants were cultivated in hydroponic culture for 12 days on 1mM nitrate, followed by 3 days of starvation (1mM KCl). 5 min 15N labeling was performed with plants directly from the KCl condition (t0) or after resupply 2h or 4h with nitrate before the labeling. NO3 influx was assayed as described by Delhon et al. (1995)[S2] using 1mM $K^{15}NO3$ (99 atom% excess 15N) in the hydroponic solution. Roots were then dried at 70°C for 72 h, and about 1.5 mg of dry weight was analyzed for total N and atom%. The 15N content of the samples was measured using isotope ratio mass spectrometry (EA Isolink CN IRMS System, ThermoFisher Scientific, Japan).

Exogenous tZ application

The plants were grown on Petri dishes containing the same media as hydroponic culture, supplemented with 0.8% agar. The 10-day-old plants analyzed were first cultivated for 7 days on 1mM KNO3 and then transferred 3 days to new Petri dishes containing 0.01% Dimethyl sulfoxide (DMSO) with either 1mM KNO3 or KCL (Mock), or KCl supplemented with 25 or 50nM of tZ (Wako, Osaka, Japan)

Cortical cells counting

Samples for observation of root meristem were collected and mounted in chloral hydrate for overnight clearing. The meristematic cortical cell file was defined by the number of cells from the initials to the increase in cell size by 2, as previously described by (Ioio et al., 2007)[S3] and illustrated in Supplemental Figure 8.

Microscopy of *IPT3 and IPT5* reporter lines

The profile of IPT3 protein level during nitrate fluctuation was addressed using plants expressing the construction *proIPT3::IPT3(cds)-RFP* in *ipt3,5,7* triple mutant background called IPT3-RFP (see main text material and method for line construction). Plants' growth conditions were similar to those illustrated in Figure 1A but using Petri dishes. As for hydroponic culture, the plants were transferred in new KNO3 Petri dishes to ensure nitrate concentration after 7- and 11-days post-germination (24h before observation). The

fluorescence intensity of IPT3-RFP was measured from confocal *z*-series pictures (Olympus FV1000-D BX61, software Olympus fluoview V4.2b, laser 559 at 5% and RFP filter), obtained in the three different regions of each root, and quantified using ImageJ. Among Z-stack pictures, we selected the best focus for each cell signal (spot) based on the spot circularity and not on brightness criteria. For each selected focus, the same rectangular area (42.421) was employed to quantify the focused spot of the RFP signal (Supplemental Figure 3D-E) as well as the surrounding signal between spots for background noise estimation. CTCF was calculated as [Integrated Density – (Area of selected cell x Mean fluorescence of the background)]. Here, we didn't consider the effect of the treatment on the spot numbers but only on the intensity of an existing signal, meaning that the level of RFP signal (IPT3 protein) is probably overestimated, especially during starvation, where spot numbers seemed to decrease (not quantified).

For optical sectioning (XZ) the roots were first fixed in 4% paraformaldehyde in phosphate buffer for 1h under vacuum, washed 2 times with 100mM phosphate buffer and placed in Clearsee solution (Kurihara et al., 2015)[S4] containing 2mM calcofluor white for 40 minutes. After at least 30 min wash in Clearsee, roots were mounted in 50% glycerol for observation under inverted confocal (Olympus FV3000, software FV31S-SW). Calcofluor was excited with laser 405 (0.3 to 2% depending on the root region and staining) and RFP with laser 559 at 6%. A series of 156 to 163 Z stacks with 0.6µm intervals was performed for each picture subjected to optical cross section. The merged pictures between RFP and calcofluor white were performed using ImageJ and cross section using the orthogonal view.

Both construction *pIPT3::GFP* and *pIPT5::GFP* have been previously characterized (Takei et al., 2004)[S5]*.* They were employed in Supplemental Figure 17 to investigate differences between *IPT3* and *IPT5* expression patterns during growth in a constant and fluctuating nitrate environment. For each time point represented in Supplemental Figure 5, 5 plants were observed by Olympus BX51 epifluorescent microscope (camera Olympus D72 and cellSens Standard software), and representative pictures were used for the figure.

To investigate whether *clf-29* mutation modifies the *IPT3* tissue expression profile, we introduced, by crosses, the *clf-29* background in plants expressing the construction *pIPT3::GFP (Takei et al., 2004)*[S5]*.* Then*,* 12-day-old plants cultivated in Petri dishes containing 1mM KNO3 (renewal as previously explained) were observed by Olympus BX51 epifluorescent microscope.

Additional ChIP experiments

ChIP experiments presented in the supplemental were performed as explained in the main text; we will specify here the antibody used. Chromatin was precipitated with antibodies against H3 (2.5 µg *Abcam* 1791), and immunoprecipitation of CFP-CLF was performed overnight using 20ul of GFP-Trap MA (*Chromotek*) previously washed 3 times with ChIP dilution buffer as for ChIP protocol.

Supplemental references:

[S1] **Inskeep, W. P., and Bloom, P. R.** (1985). Extinction Coefficients of Chlorophyll *a* and *b* in *N, N*-Dimethylformamide and 80% Acetone. *Plant Physiol.* **77**:483–485.

[S2] **Delhon, P., Gojon, A., Tillard, P., and Passama, L.** (1995). Diurnal regulation of NO3− uptake in soybean plants I. Changes in NO3− influx, efflux, and N utilization in the plant during the day/night cycle. *J. Exp. Bot.* **46**:1585–1594.

[S3] **Ioio, R. D., Linhares, F. S., Scacchi, E., Casamitjana-Martinez, E., Heidstra, R., Costantino, P., and Sabatini, S.** (2007). Cytokinins Determine Arabidopsis Root-Meristem Size by Controlling Cell Differentiation. *Curr. Biol.* **17**:678–682.

[S4] **Kurihara, D., Mizuta, Y., Sato, Y., and Higashiyama, T.** (2015). ClearSee: a rapid optical clearing reagent for whole-plant fluorescence imaging. *Dev. Camb. Engl.* **142**:4168–4179.

[S5] **Takei, K., Ueda, N., Aoki, K., Kuromori, T., Hirayama, T., Shinozaki, K., Yamaya, T., and Sakakibara, H.** (2004). AtIPT3 is a Key Determinant of Nitrate-Dependent Cytokinin Biosynthesis in Arabidopsis. *Plant Cell Physiol.* **45**:1053–1062.
